# The Global Response Regulator RegR Controls Expression of Denitrification Genes in *Bradyrhizobium japonicum*

**DOI:** 10.1371/journal.pone.0099011

**Published:** 2014-06-20

**Authors:** Maria J. Torres, Montserrat Argandoña, Carmen Vargas, Eulogio J. Bedmar, Hans-Martin Fischer, Socorro Mesa, María J. Delgado

**Affiliations:** 1 Estación Experimental del Zaidin, Consejo Superior de Investigaciones Científicas (CSIC), Granada, Spain; 2 Departamento de Microbiología y Parasitología, Universidad de Sevilla, Sevilla, Spain; 3 ETH Zurich, Institute of Microbiology, Zurich, Switzerland; Niels Bohr Institute, Denmark

## Abstract

*Bradyrhizobium japonicum* RegSR regulatory proteins belong to the family of two-component regulatory systems, and orthologs are present in many Proteobacteria where they globally control gene expression mostly in a redox-responsive manner. In this work, we have performed a transcriptional profiling of wild-type and *regR* mutant cells grown under anoxic denitrifying conditions. The comparative analyses of wild-type and *regR* strains revealed that almost 620 genes induced in the wild type under denitrifying conditions were regulated (directly or indirectly) by RegR, pointing out the important role of this protein as a global regulator of denitrification. Genes controlled by RegR included *nor* and *nos* structural genes encoding nitric oxide and nitrous oxide reductase, respectively, genes encoding electron transport proteins such as *cycA* (blr7544) or *cy*_2_ (bll2388), and genes involved in nitric oxide detoxification (blr2806-09) and copper homeostasis (*copCAB*), as well as two regulatory genes (bll3466, bll4130). Purified RegR interacted with the promoters of *norC* (blr3214), *nosR* (blr0314), a *fixK*-like gene (bll3466), and bll4130, which encodes a LysR-type regulator. By using fluorescently labeled oligonucleotide extension (FLOE), we were able to identify two transcriptional start sites located at about 35 (P1) and 22 (P2) bp upstream of the putative translational start codon of *norC*. P1 matched with the previously mapped 5′end of *norC* mRNA which we demonstrate in this work to be under FixK_2_ control. P2 is a start site modulated by RegR and specific for anoxic conditions. Moreover, qRT-PCR experiments, expression studies with a *norC-lacZ* fusion, and heme *c*-staining analyses revealed that anoxia and nitrate are required for RegR-dependent induction of *nor* genes, and that this control is independent of the sensor protein RegS.

## Introduction

The *Rhizobiales* order of α-Proteobacteria include Gram-negative nitrogen-fixing soil bacteria collectively named rhizobia which have the unique ability to establish N_2_-fixing symbioses with legume roots and stems of some aquatic legumes, leading to the formation of new plant organs called nodules. Expression of nitrogen fixation and other symbiosis-related genes requires low-oxygen conditions [1]–[2]. To cope with oxygen limitation prevailing under microoxic free-living conditions or in so-called bacteroids existing within plant cells of nodules, rhizobial species express the high-affinity *cbb*_3_ oxidase encoded by the *fixNOQP* operon [3]. Moreover, some rhizobial species are able to use nitrate as final electron acceptor to support respiration under microoxic or anoxic conditions [4]–[6]. The switch from oxygen to nitrate respiration leads to a reduction in the ATP yield, yet it allows bacteria to survive and multiply under oxygen-limiting conditions [7]. Denitrification has been defined as the dissimilatory reduction of nitrate (NO_3_^−^) or nitrite (NO_2_^−^) to N_2_ via the gaseous intermediates nitric oxide (NO) and nitrous oxide (N_2_O) with concomitant ATP generation [8]. This process requires four separate enzymatic reactions catalyzed by nitrate-, nitrite-, nitric oxide-, and nitrous oxide reductases, encoded by *nar*/*nap*, *nir*, *nor*, and *nos* genes, respectively [9]–[11].

In recent years, it has emerged that many rhizobial species have denitrification genes [4]–[6]. Among them, the soybean symbiont *Bradyrhizobium japonicum* is considered the model organism for studying rhizobial denitrification. In this bacterium, denitrification depends on the *napEDABC*, *nirK*, *norCBQD*, and *nosRZDYFLX* genes that encode a periplasmic nitrate reductase (Nap), a copper-containing nitrite reductase (NirK), a *c*-type nitric oxide reductase (cNor), a nitrous oxide reductase (Nos), respectively [4]. Similar to many other denitrifiers, expression of denitrification genes in *B. japonicum* requires both oxygen limitation and the presence of nitrate or a derived nitrogen oxide [4]. Perception and transduction of the “low-oxygen signal” are mediated by conserved regulatory proteins that are integrated into species-specific networks in different rhizobia [1]–[2]. Two interlinked oxygen responsive regulatory cascades are present in *B. japonicum*, the FixLJ-FixK_2_ and the RegSR-NifA cascades [12]. A moderate decrease in the oxygen concentration in the gas phase (≤5%) is sufficient to activate expression of FixLJ-FixK_2_-dependent targets [12]. This “low-oxygen” signal is sensed by the heme-based sensory kinase FixL which auto-phosphorylates and transfers the phosphoryl group to the FixJ response regulator which then activates transcription of *fixK_2_*. In turn, FixK_2_ induces expression of regulatory genes such as *rpoN*_1_, *fixK*_1_, *nnrR* [13]–[15], genes associated with microoxic metabolism, e.g. *fixNOQP* [14]–[16] as well as genes involved in denitrification such as *nap*, *nirK*, *nor*, and *nos* [4], [13]–[14], [17]. Induction of genes controlled by the RegSR-NifA cascade requires very low oxygen concentration (≤0.5%) because of the pronounced oxygen sensitivity of NifA. The response regulator RegR of the RegSR two-component regulatory system induces expression of the *fixR-nifA* operon which is preceded by two overlapping promoters, P1 and P2 [18]–[20]. RegR activates transcription originating from P2 under all oxygen conditions via binding to a DNA element located around position −67 upstream of the transcription start site. Upon a switch to low-oxygen or anoxic conditions, the redox-responsive NifA protein in concert with RNA polymerase containing RpoN (σ^54^) enhances its own synthesis via activation of the −24/−12-type promoter P1. In *B. japonicum*, RpoN is encoded by the two highly similar and functionally equivalent genes (*rpoN*_1_ and *rpoN*_2_) [21]. Since *rpoN*_1_ is under the control of FixK_2_, this gene represents the link between the two regulatory cascades. Targets of NifA include *nif* and *fix* genes, which are directly or indirectly involved in nitrogen fixation, and also genes that are not essential for this process or have an unknown function [1], [22]–[23]. Recent results from our group showed that NifA is also required for maximal expression of *nap*, *nirK*, and *nor* genes, suggesting a new role for the RegSR-NifA regulatory cascade in the control of the denitrification genes in *B. japonicum* [24]. A large number of members of the RegR regulon have been identified by transcriptome analysis of a *B. japonicum regR* mutant grown under oxic and microoxic free-living conditions and also in bacteroids [25]. However, no data are available about the RegR regulon in cells grown under denitrifying conditions. Moreover, we have recently demonstrated that the level of NorC is significantly lower in membranes isolated from a *B. japonicum regR* mutant compared to the wild type when cells were grown under denitrifying conditions [26]. However, the involvement of RegSR in *norCBQD* genes expression has not been investigated so far.

Here, we have performed a comparative transcriptome analysis of *B. japonicum* wild type and a *regR* mutant grown under denitrifying conditions. Among the novel RegR targets, *nor* genes encoding the nitric oxide reductase were identified. By different approaches we also demonstrated that RegR control of *nor* genes induction requires anoxia and nitrate, and that this control is independent on the sensor protein RegS.

## Materials and Methods

### Bacterial Strains and Growth Conditions

The wild-type strain *B. japonicum* 110*spc*4 [27] and its derivatives 2426 (Δ*regR*), 2409 (Δ*regS*), [20], and 9043 (Δ*fixK*_2_) [15] were used in this study. Strain 2499 [13] is *B. japonicum* 110*spc*4 containing a *norC–lacZ* fusion. In this work, plasmid pRJ2499 containing the *norC–lacZ* fusion [13], was integrated by homologous recombination into the chromosome of the *regR* and *regS* mutant strains resulting in strains 2499RR and 2499RS, respectively. *B. japonicum* strains were grown oxically at 30°C in peptone-salts-yeast extract (PSY) medium supplemented with 0.1% L-arabinose [14], [27]. Growth under oxygen-limiting conditions was performed in Bergersen minimal medium [28] with succinate as carbon source and supplemented (BSN) or not (BS) with 10 mM KNO_3_. For comparison with previous experiments, yeast extract-mannitol (YEM) medium [29] supplemented with 10 mM KNO_3_ was used for anoxic cultures of the wild type and *fixK_2_* mutant in some primer extension experiments. Once they were inoculated to an OD_600_ of about 0.2, cultures were subjected to oxygen-limiting conditions generated by two different experimental procedures. In the first set of experiments, 17 ml serum tubes or 500 ml flasks containing 5 or 200 ml medium, respectively, were sealed with rubber septa stoppers and the headspace atmosphere was replaced by a gas mixture (2% oxygen, 98% argon) before cultures were incubated. In the second set, cells were incubated in completely filled, sealed 200 ml bottles or 17 ml tubes without gas exchange. The latter conditions are referred to as anoxic conditions throughout the manuscript. Antibiotics were added to *B. japonicum* cultures at the following concentrations (µg ml^−1^): cloramphenicol 20, spectinomycin 200, streptomycin 100, tetracycline 100. *Escherichia coli* strains were cultured in Luria-Bertani (LB) medium [30] at 37°C. *E. coli* S17-1 [31] served as the donor for conjugative plasmid transfer. Tetracycline was used at 10 µg ml^−1^ in *E. coli* cultures.

### RNA Isolation, cDNA Synthesis, and Microarray Analysis

Cultures of *B. japonicum* wild type and *regR* mutant strains were grown anoxically in BSN medium to an OD_600_ of about 0.4. Cell harvest, isolation of total RNA, cDNA synthesis, fragmentation, labeling and conditions for microarray hybridization were done as described previously [22], [25], [32]–[33]. Details of the custom designed Affymetrix *B. japonicum* gene chip BJAPETHa520090 (Santa Clara, CA) have also been described previously [22]. For each strain, a minimum of four biological replicates was analyzed. Details on data processing, normalization, and further analysis are described elsewhere [33]. GeneSpring GX 7.3.1 software (Agilent Technologies, Santa Clara, CA) was used for comparative analyses. Only the probe sets that were called “present” or “marginal” in ≥75% of the replicates of each experiment were considered for further analysis. The student *t*-test with a P value threshold of 0.025 was applied for statistical comparisons. We considered genes passing the statistical tests as differentially expressed only if the relative change in expression (*n*-fold) was ≥2 or ≤−2 when different conditions or strains were compared. Operon predictions were done as described in Hauser et al. (2007) [22] and Mesa et al. (2008) [14]. An operon-like organization of genes (bicistronic or larger) was assumed if they were orientated in the same direction and separated by less than 32 bp. This distance was enlarged to 100 bp if the first three letters in the gene names were identical.

### Quantitative Real-Time PCR

Expression of *nosZ, nosY, norC*, blr2808, *napE, napA, cycA, copC*, bll3466, bll4130 and bll2388 genes was also analyzed by quantitative reverse transcription-PCR (qRT-PCR) using an iQ™5 Optical System (Bio-Rad, CA). *B. japonicum* wild-type and *regR* cultures, RNA isolation and cDNA synthesis were performed as described for microarray experiments. Primers for the PCR reactions (Table S1 in supplemental material) were designed with Primer3Web v.0.4.0 (http://frodo.wi.mit.edu/primer3/input.htm) to have a melting temperature of 57°C to 62°C and generate PCR products of 50 to 100 bp. Each PCR reaction contained 9.5 µl of iQ™ SYBR Green Supermix (Bio-Rad), 2 µM (final concentration) of individual primers and appropriate dilutions of different cDNAs in a total volume of 19 µl. Reactions were run in triplicates. Melting curves were generated to verify the specificity of the amplification. Relative changes in gene expression were calculated as described elsewhere [34]. Expression of the primary sigma factor gene *sigA* was used as a reference for normalization (primers SigA-1069F and SigA-1155R; [25]).

### Heme-Staining Analysis

Cells of *B. japonicum* grown oxically in 150 ml PSY medium were harvested by centrifugation at 8,000× *g* for 5 min, washed twice with BS or BSN, resuspended in 500 ml of the same medium, and cultured under anoxic conditions or with 2% initial O_2_ concentration for 48 hours (final resulting OD_600_ about 0.5). Cells were disrupted using a French pressure cell (SLM Aminco, Jessup, MD, USA) and membranes were isolated as described previously [35]. Membrane protein aliquots were diluted in sample buffer [124 mM Tris-HCl, pH 7.0, 20% glycerol, 4.6% sodium dodecyl sulfate (SDS) and 50 mM 2-mercaptoethanol], and incubated at room temperature for 10 min. Membrane proteins were separated at 4°C in 12%-SDS polyacrylamide gel electrophoresis (20 µg protein per lane), transferred to a nitrocellulose membrane and stained for heme-dependent peroxidase activity as described previously [36] using the chemiluminescence detection kit “SuperSignal” (Pierce, Thermo Fisher Scientific, IL, USA).

### NO Consumption Activity

Cells of *B. japonicum* were cultured anoxically during 48 hours in BSN (OD_600_ of about 0.5). Cells were harvested by centrifugation at 8,000× *g* for 10 min at 4°C, and washed with 50 mM Tris-HCl buffer (pH 7.5). NO consumption rates were determined with a 2 mm ISONOP NO electrode APOLO 4000 (World Precision Inst., Sarasota, FL) in a 2 ml temperature-controlled, magnetically stirred reaction chamber [37]. The membrane-covered electrode was situated at the bottom of the chamber above the stirrer and reactants were injected with a Hamilton syringe through the port in the glass stopper. The chamber was filled with 760 µl of 25 mM phosphate buffer (pH 7.4), 900 µl of cell suspension (4–5 mg protein), 100 µl of an enzyme mix of *Aspergillus niger* glucose oxidase (40 units ml^−1^) and bovine liver catalase (250 units ml^−1^) (Sigma-Aldrich, St. Louis, MO), 90 µl 1 M sodium succinate, and 100 µl of 320 mM glucose. Once a steady base line was observed, 50 µl of a saturated NO solution (1.91 mM at 20°C) was added to the cuvette to start the reaction. Each assay was run until the NO detection had dropped to zero, *i.e.* when all NO was oxidized.

### β-Galactosidase Assays

To measure β-galactosidase activity, strains 2499, 2499RR and 2499RS were grown oxically in PSY medium, collected by centrifugation at 8,000× *g* for 10 min at 4°C, washed twice with BS or BSN medium and cultured anoxically or under 2% initial O_2_ in the same medium for 48 h (OD_600_ of about 0.5). Activity was determined with permeabilized cells from at least three independently grown cultures assayed in triplicate for each strain and condition. β-Galactosidase assays were performed essentially as previously described [30]. The absorbance data for *A*_420_, *A*_550_, and *A*_600_ were determined for all samples in a plate reader (SUNRISE Absorbance Reader, TECAN, Männedorf, Switzerland) using the XFluor4 software (TECAN). Data were then transferred to Microsoft Excel to calculate the specific activities in Miller units.

### Fluorescently Labeled Oligonucleotide Extension (FLOE)

*B. japonicum* wild type, *regR* and *fixK_2_* mutant strains were cultured as indicated above for microarray and qRT-PCR experiments. Cells were harvested and total RNA was isolated using the hot phenol extraction procedure described by Babst and coworkers (1996) [38]. To determine the transcription start site of *norC* gene, the NorC53 reverse primer was synthesized, HPLC-purified, and labeled with 6-carboxyfluorescein (6-FAM) at the 5′-end (Eurofins MWG Operon, Ebersberg, Germany). The sequence of NorC53 was 5′-GAGCCGCCGTAGAAGACGTTTC-3′ which corresponds to positions 53-31 downstream of the annotated translation start codon of *norC*. The primer extension assay was performed using Avian Myeloblastosis Virus Reverse Transcriptase (AMV RT; Promega, Wisconsin, USA). The reaction mixture (50 µl) contained MgCl_2_ (5 mM), dNTPs (2 mM each), AMV RT 1X buffer, 50 pmoles FAM-labeled primer, 7–10 µg of RNA and water. Reactions were incubated at 65°C for 15 minutes, then 2 µl of the AMV/RNasin mixture (1∶1; Promega) was added. Next, the mixtures were kept at 15°C for 10 minutes followed by incubation at 45°C and 95°C for 45 and 5 minutes, respectively. Finally, 2 µl of RNase (2 mg ml^−1^) was added to the reactions which were further incubated at 37°C for 90 minutes. The length of the reverse transcribed cDNA products was analyzed in an ABI Prism 3100 Genetic Analyzer capillary electrophoresis instrument (Newbiotechnic S.A., Seville, Spain). GeneScan version 3.1.2 (Applied Biosystems, Austin TX) was used to analyze the data, *i.e.* peak identification and determination of the length and abundance of cDNA.

### Electrophoretic Mobility Shift Assays (EMSAs)

Binding of RegR to putative target promoters was tested by EMSA using radiolabeled PCR fragments obtained with the primers listed in Table S1. PCR fragments were end labeled with [γ-^32^P] ATP using T4 polynucleotide kinase (MBI Fermentas), and subsequently purified over Micro Bio-Spin 6 chromatography columns (Bio-Rad, Spain). His-tagged RegR was overexpressed and purified as described previously [39]. For *in vitro* phosphorylation, RegR protein (40 µM final concentration) was incubated with 25 mM acetyl phosphate (Sigma-Aldrich, St. Louis, MO) in DNA binding buffer [20] for 1 h at 30°C. Phosphorylated RegR (0 to 7.5 µM) was incubated with column-purified DNA fragments (0.5 to 1 µg) in DNA binding buffer in a total volume of 20 µl. After 15-min incubation at 30°C, samples were mixed with loading dye and separated on 8% non-denaturing polyacrylamide gels in Tris-borate 89 mM and EDTA 2 mM electrophoresis buffer pH 8.2 for 2 h at 70 V. Gels were dried, and radiolabeled bands were visualized with a phosphorimager (Bio-Rad, Spain).

### Microarray Data Accession Number

The microarray data are available in the NCBI Gene Expression Omnibus database (GEO; http://www.ncbi.nlm.nih.gov/geo) under GEO Series accession number GSE56668.

## Results

### Transcriptional Profiling of a *B. japonicum regR* Mutant Grown Under Free-living Denitrifying Conditions

**Figure 1:**
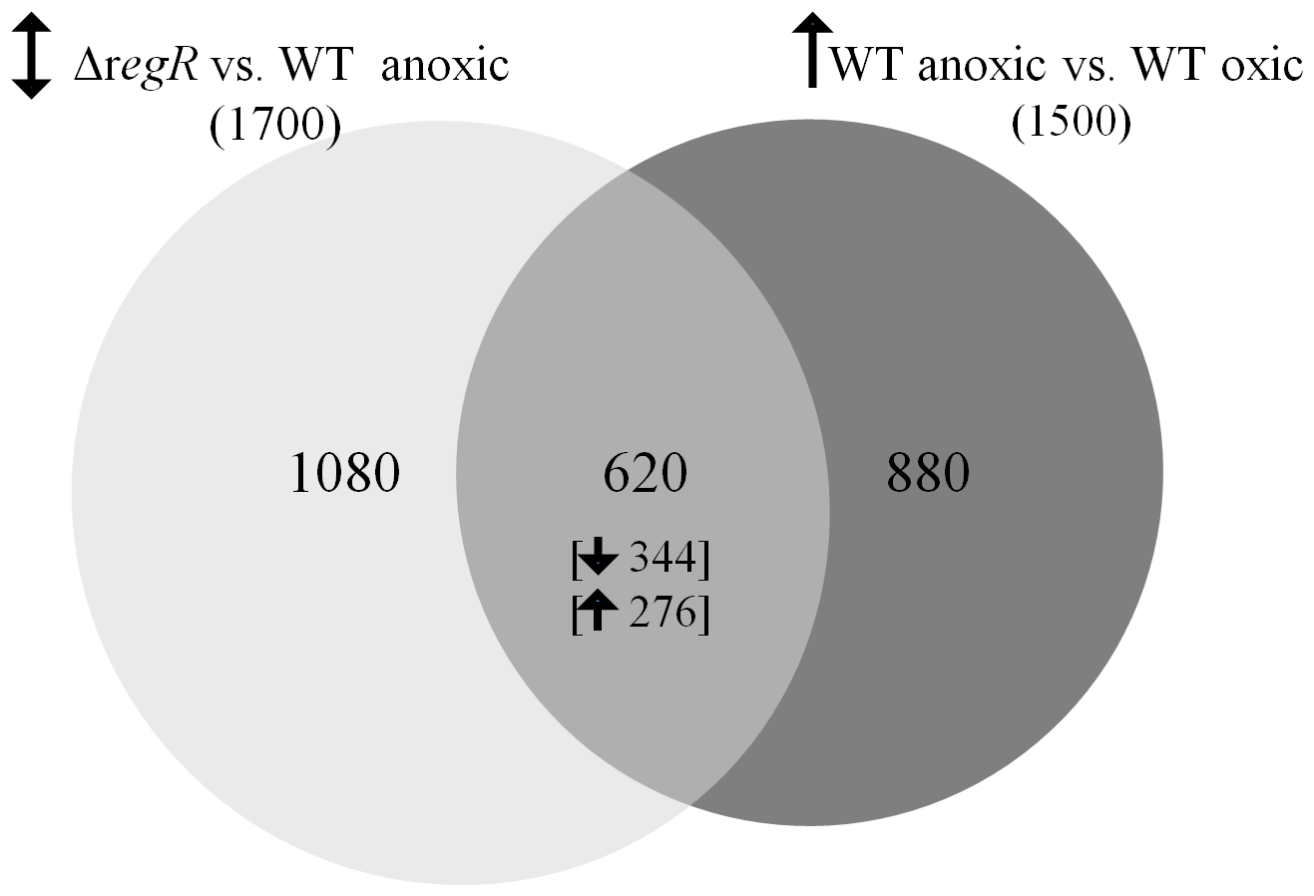
Venn diagram based on anoxically induced genes of the *B. japonicum* wild type (WT; dark grey circle) and genes which, when compared with the wild type, are differentially expressed in the *regR* mutant both grown under anoxic denitrifying conditions (light grey circle). The overlap shows the fraction of genes which are induced in the wild type and differentially transcribed in the *regR* mutant (the number of genes upregulated or downregulated in the *regR* mutant appears in brackets). Strains and conditions are indicated alongside the circles. Up-down arrows reflect increased and decreased gene expression in microarray analyses. Numbers in parentheses indicate the total number of differentially expressed genes.

Comparative analyses of *B. japonicum* wild type and the *regR* mutant strain grown under anoxic conditions in BSN medium revealed that approximately 1,700 genes were differentially expressed in the *regR* mutant (Fig. 1). The main focus of this work was the identification of genes that were upregulated in the wild type under denitrifying conditions (in comparison with oxically-grown wild type) [22], [33], and at the same time regulated by RegR. The comparison of both regulons gave a total number of 620 genes (Fig. 1, Table S2 in supplemental material). Within this group, we focused on the genes positively controlled by RegR under denitrifying conditions (344 genes). Among them, we found genes involved in the denitrification process, such as *nosRZDFYLX* encoding nitrous oxide reductase [40], and *norECBQD* genes encoding nitric oxide reductase [41]. We also identified *cycA* which codes for cytochrome *c*_550_ that is implicated in electron delivery to NirK, the Cu-containing nitrite reductase [42]–[43]. Of the *napEDABC* genes specifying periplasmic nitrate reductase [35], only *napB* and *napC* genes were identified as RegR dependently transcribed, yet the relative fold change (FC) of expression was only −2.8 and −3.4, respectively (Table S2). Surprisingly, *nirK* could not be identified among the RegR-controlled genes (Table S2). We also found as RegR target the *cy_2_* gene (bll2388) that encodes the previously identified FixK_2_-dependent cytochrome *c*_2_ [14], which suggests that this gene might be relevant for life under denitrifying conditions. In addition to denitrification genes, numerous other genes were identified as candidates for being RegR targets under anoxic conditions (Table S2).

**Figure 2:**
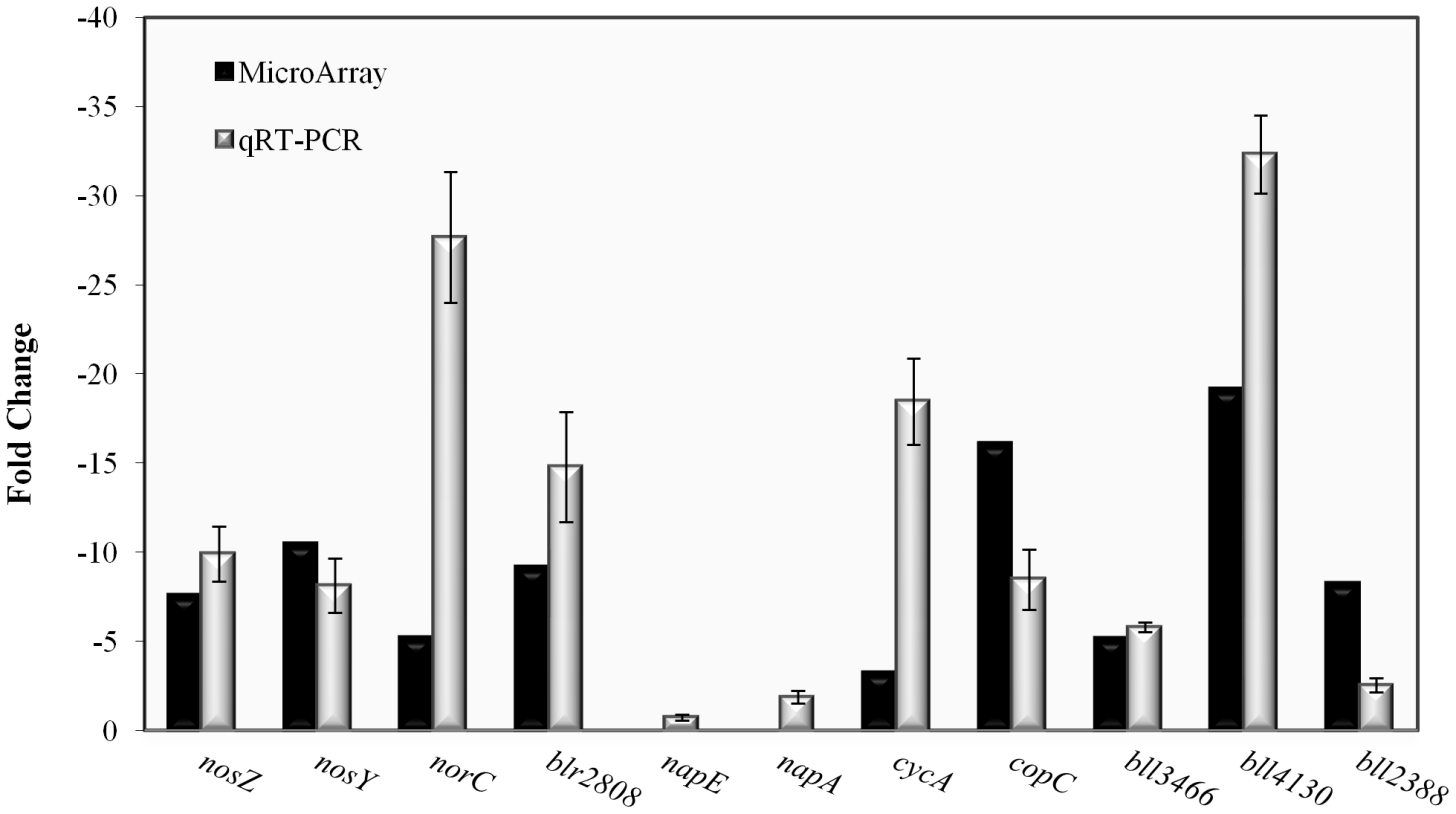
Comparison of expression data generated by microarray experiments (black bars) and qRT-PCR (grey bars). RNA was isolated from the wild-type strain and the *regR* mutant cultured in BSN medium under anoxic conditions. qRT-PCRs were repeated in three independent experiments each including four parallel amplification reactions. Fold-change values refer to differences of expression when the *regR* mutant was compared with the wild type.

To validate the microarrays results, we performed qRT-PCR with several selected genes. Among them, we have selected genes encoding (i) denitrification enzymes (*nosZ*, *nosY*, *norC*, *napE* and *napA*) and electron transfer proteins (*cycA*, bll2388), (ii) NO detoxification proteins (blr2806-09), (iii) copper homeostasis proteins (*copC*), and (iv) transcription factors (bll3466 encoding a FixK-like protein and bll4130 which encodes a LysR-type regulator). As shown in Fig. 2, we confirmed by qRT-PCR RegR-dependent induction of genes for nitrous oxide reductase (*nosZ*, *nosY*), nitric oxide reductase (*norC*), and also *cycA* and bll2388. Likewise, qRT-PCR confirmed the microarray data which indicated that genes encoding the periplasmic nitrate reductase (*napE* and *napA*) are not significantly controlled by RegR. Furthermore, we could verify RegR-dependent expression of blr2808 which is part of a gene cluster (blr2806-blr2809) possibly involved in NO detoxification and nitrate assimilation [44]. Finally, qRT-PCR also confirmed RegR-dependent expression of *copC*, bll3466, and bll4130 (Fig. 2). Note that differences observed between FC values determined by microarray technology and qRT-PCR such as those observed for *norC*, *cycA* and bll2388 are not uncommon.

### RegR Binding to the Promoter Region of New Target Genes

**Figure 3:**
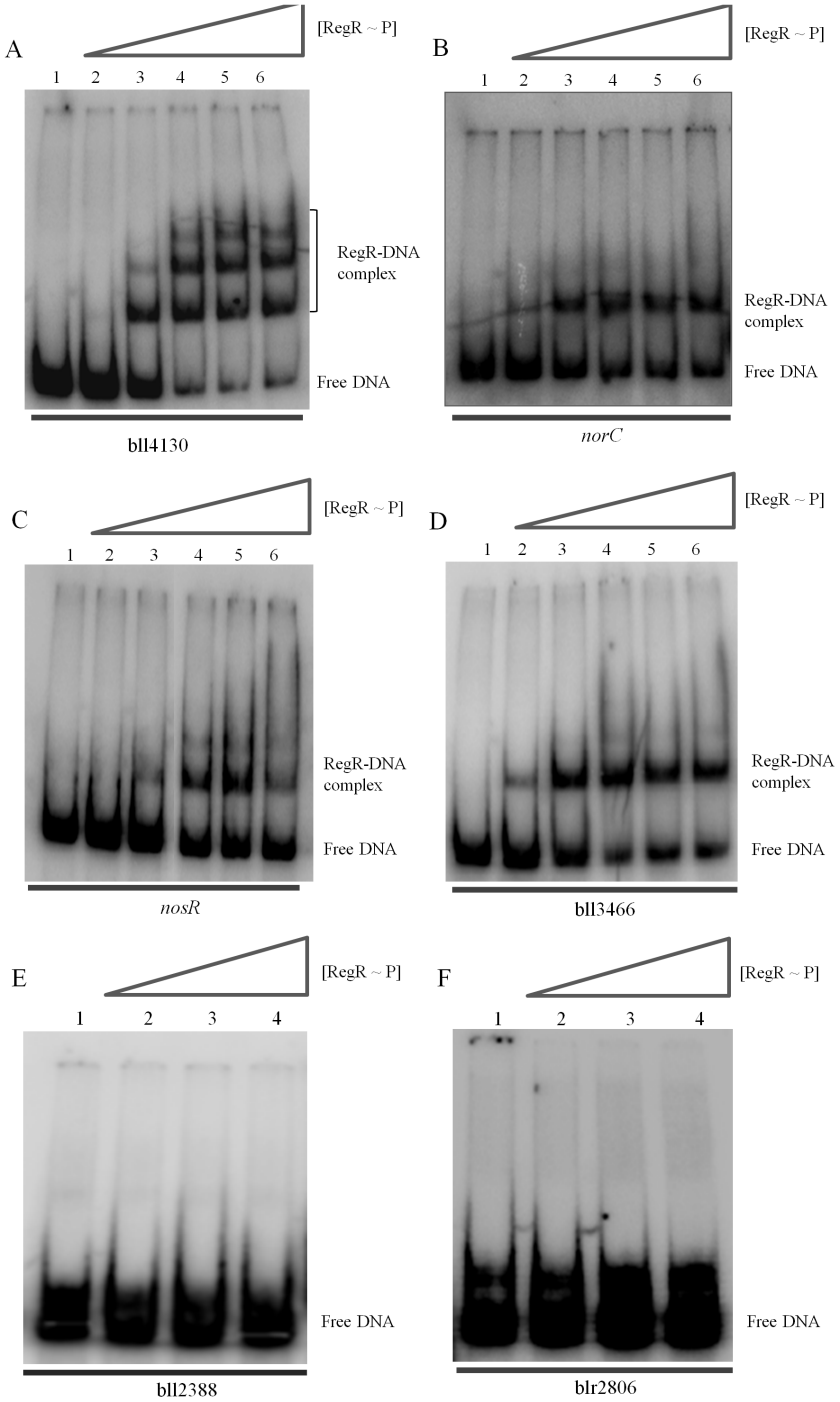
Analysis of RegR binding to the promoter region of several putative target genes by EMSA. Increasing amounts of purified RegR∼P were incubated with constant amounts (0.5 to 1 µg) of double-stranded ^32^P-labeled PCR amplified products from the promoter region of *B. japonicum* bll4130, *norC*, *nosR*, bll3466, bll2388 and blr2806 genes. In panels A to D, RegR concentrations were 1.5 µM (lanes 2), 3 µM (lanes 3), 4.5 µM (lanes 4), 6 µM (lanes 5) and 7.5 µM (lanes 6). In panels E and F, RegR concentrations were 0.8 µM (lanes 2), 1.6 µM (lanes 3) and 3.2 µM (lanes 4). No RegR protein was added to the control reactions loaded in lane 1 of all panels. Samples were run on 6% non-denaturing polyacrylamide gels and visualized with a phosphorimager.

Because microarray analysis cannot discriminate directly and indirectly controlled genes, we performed DNA binding analyses by EMSA to identify direct RegR target genes. We used PCR fragments corresponding to the promoter region of six candidate genes whose expression was found to be under positive control of RegR (Table 1). ^32^P-labeled fragments were incubated with increasing concentrations of phosphorylated RegR (0 to 7.5 µM). As positive and negative controls, we used amplification products covering the promoter and the coding region of the bll2087 gene, respectively (Table S1) [32]. RegR displayed consistent binding to the promoter region of bll4130 (Fig. 3A). In addition to bl4130, RegR bound to the DNA probes derived from the promoter regions of *norC* (Fig. 3B), *nosR* (Fig. 3C), and bll3466 (Fig. 3D). By contrast, no binding was observed to the promoter regions of bll2388 (Fig. 3E) and blr2806 (Fig. 3F). While EMSAs with the *norC*, *nosR* and bll3466 promoter regions showed one predominat retarded band (Fig. 3B, C and D), up to 3 bands of distinct mobility were observed with the bll4130 promoter and increasing RegR concentrations (Fig. 3A).

**Table 1:** Summary of RegR binding studies with promoter regions of anoxically induced, RegR-controlled *B. japonicum* genes.

Gene no.^a^	Gene name^b^	Description^c^	Genomic region^d^	Shift^e^	Putative RegR-box position^f^	Sequence of putative RegR-box^g^
blr0314	*nosR*	Nitrous oxide reductase expression regulator	−195 to +46	+	−73	T**G**C**G**T**C**AAC**G**G**C**GA
					−39	C**G**C**G**G**C**CCG**G**T**C**GG
bll2388	*cy_2_*	Cytochrome *c_2_*	−143 to +31	−		None found
blr2806		Nitrite extrusion protein	−214 to +34	−	−64	C**G**C**G**C**C**TCCGT**G**G**C**CG
					−49	G**G**A**G**G**C**AGA**G**C**C**TG
blr3214	*norC*	Nitric oxide reductase subunit C	−149 to +53	+	−64	C**G**C**G**CGAAGC**G**G**C**
					−123	C**G**T**G**T**C**GGCC**G**T**C**GT
bll3466	*fixK*	Transcriptional regulator FixK-type	−160 to +58	+	−78	T**G**C**G**A**C**ATC**G**G**C**GGC
					−88	C**G**A**G**C**C**GGAGT**G**CGAC
bll4130		Transcriptional regulatory protein LysR-family	−114 to +61	+	−51	T**G**C**G**G**C**TTTC**G**TGCC
					−98	T**G**C**G**G**C**AAAG**G**AGCC

a All listed genes are differentially expressed (FC<−2) in a comparison of the wild type with the Δ*regR* mutant both grown under the anoxic, denitrifying conditions as described in this work.

b Gene name as indicated in the EMBL-EBI database.

c Protein description according to Kaneko and coworkers, 2002 [67].

d Genomic region included in the PCR fragment used for EMSAs. Coordinates refer to the first nucleotide position relative to the annotated translation start site of the genes listed in column 1.

e Indicates qualitatively whether (+) or not (−) RegR binding was observed to the DNA fragments specified in column 4.

f Position of the 5′-end nucleotide of the putative RegR box (column 7) relative to the annotated translational start site of the associated gene.

g Sequence of the putative RegR binding sites. Conserved nucleotides are highlighted.

Inspection of DNA sequences included in the EMSA assays by using the previously proposed RegR box as query [25] allowed us to identify two putative RegR binding sites in all promoter regions except for bll2388 (Table 1). As described previously [25], half sites of the proposed RegR boxes are differently spaced and not perfectly conserved (Table 1).

### Mapping of *norC* Transcripts

**Figure 4:**
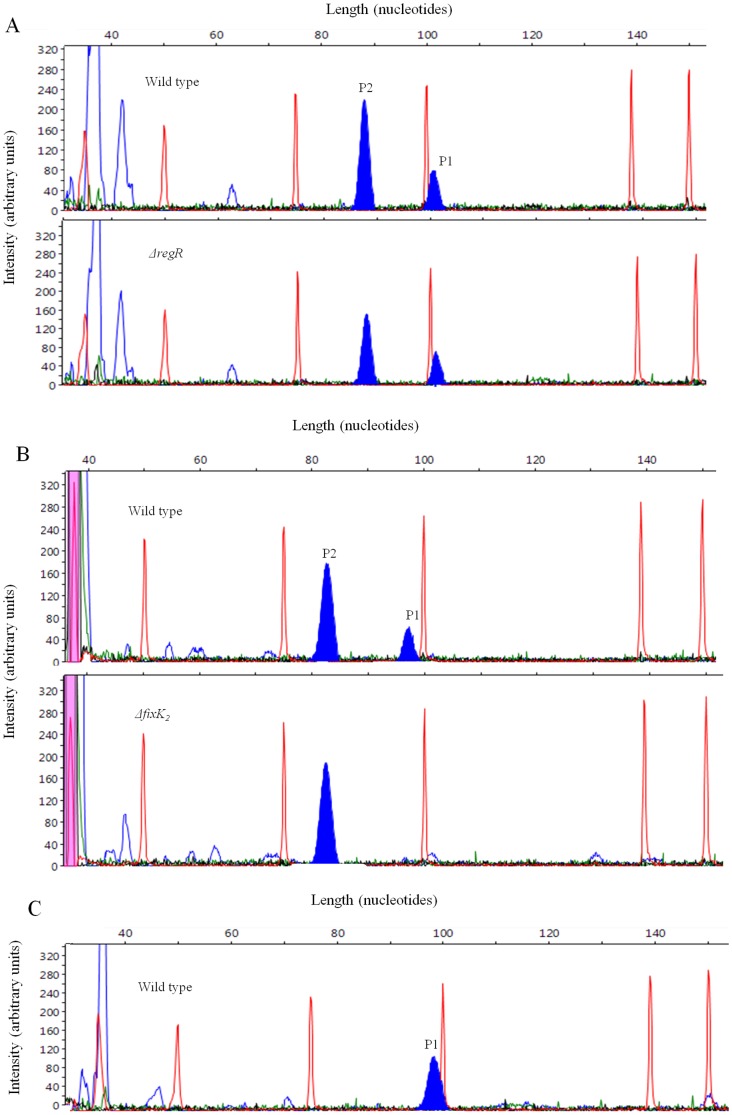
Transcription start site mapping of *B. japonicum norC* by FLOE. Panel A corresponds to FLOE electropherograms obtained by using RNA from the wild type (upper panel) and *regR* mutant (bottom panel) both cultured anoxically in BSN medium. In panel B, RNA was isolated from the wild type (upper panel) and *fixK*_2_ mutant (botton panel) both cultured anoxically in YEM medium containing 10 mM KNO_3_. In panel C, RNA was isolated from the wild type cultured in BSN medium under 2% initial O_2_. The red peaks are GeneScan-500 ROXTM internal size markers. Filled blue peaks in each panel correspond to primer extension products P1 and P2.

**Figure 5:**
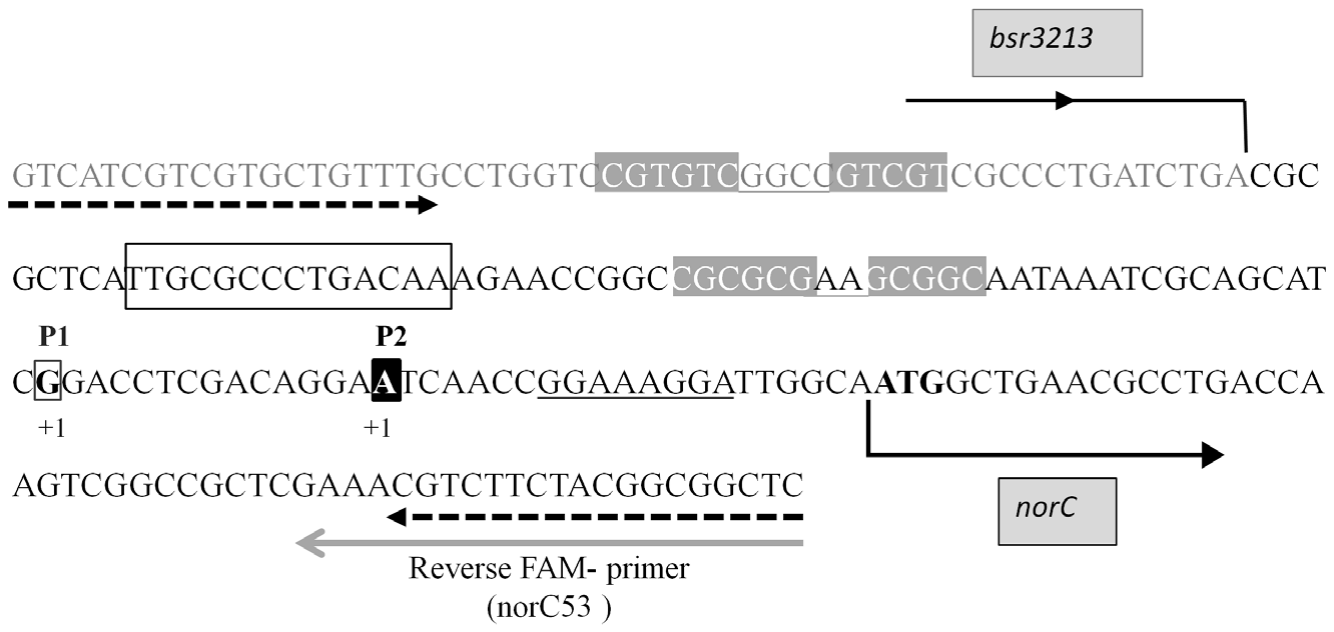
Structure of the *B. japonicum norC* promoter. Nucleotides corresponding to the start site of transcripts P1 and P2 are shown in bold, marked +1 below and highlighted with an open black box and a solid black box, respectively. The putative FixK_2_ and RegR binding sites are indicated with an open black box and two pairs of solid grey boxes, respectively. The *norC* translation start codon (ATG) as annotated in the *B. japonicum* genome database (http://kazusa.or.jp/rhizobase) is shown in bold face letters. A potential Shine-Dalgarno sequence of *norC* is underlined. Forward and reverse primers for amplification of the DNA region used in EMSAs are indicated with dashed arrows. The continuous grey arrow indicates the FAM-labeled primer used in FLOE analyses. Continuous black arrows indicate the 3′ end of bsr3213 and the 5′ end of *norC*.

In order to substantiate RegR control of *nor* genes, we mapped the transcriptional start site of *norC* in wild-type and *regR* cells grown under dentrifying conditions. Using FLOE technique we identified two signals with cDNA derived from wild-type RNA (Fig. 4, panel A, upper electropherogram) suggesting the presence of two transcriptional start sites. The cDNA signal marked as P1 corresponds to the previously proposed start site of *norC* [41] which maps to a guanosine 35 bp upstream of the translational start codon of NorC (Fig. 5). The cDNA signal designed as P2 corresponds to a second start site which maps to an adenosine located 14 bp downstream of P1 and 21 bp upstream of the *norC* start codon (Fig. 5). As the peak area is directly proportional to the number of cDNA molecules [45], the FLOE technique allows quantification of intensity of each signal in arbitrary units. The average area of P2 from 6 different replicates indicated that the abundance of P2 cDNA obtained with RNA from the *regR* mutant (Fig. 4A, lower electropherogram) was reduced by approximately 40% compared to wild type-derived cDNA (Fig. 4A, upper electropherogram). However, the area of the P1 cDNA peak did not significantly change in the *regR* mutant relative to the wild type (Fig. 4A). This suggests that RegR is needed for efficient synthesis of the P2 transcript under anoxic conditions. The transcriptional start site P1 is located 45 bp downstream of a FixK_2_ box (Fig. 5), which supports our previous notion that *norC* transcription depends on the FixK_2_ transcription factor [41]. To confirm this hypothesis, we also have performed primer extension experiments with RNA isolated from a *B. japonicum fixK_2_* mutant. As shown in Fig. 4B (lower electropherogram), the primer extension signal corresponding to P1 was absent in cDNA derived from the *fixK*_2_ mutant but present in cDNA obtained with wild-type RNA (Fig. 4B, upper electropherogram).

In other bacteria, RegSR orthologous two-component regulatory systems (e.g. RegBA, PrrBA, or ActSR) respond to different oxygen levels to adapt their respiration accordingly [46]. Therefore, we were interested to determine whether the P2 transcript proposed to be RegR-modulated under anoxic conditions is also synthesized under another oxygen-limiting condition. To do so, we performed primer extension experiments using RNA from cells grown in BSN medium and flushed with 2% oxygen at the beginning of the incubation. As shown in Fig. 4C, no P2 cDNA was detected when RNA was isolated from wild-type cells grown under these conditions. By contrast, P1 cDNA corresponding to the FixK_2_-dependent transcript was present when wild-type cells were cultured under both anoxic or 2% initial oxygen conditions (Fig. 4A, C). Similar results were obtained when we used RNA from *regR* mutant cells grown under the same conditions (data not shown). These results suggest that, in contrast to P1, P2 transcript is specific for anoxic conditions.

### RegR Control of *nor* Genes Requires Anoxia and Nitrate and is RegS Independent

In order to investigate whether or not anoxic conditions are required for RegR-dependent induction of *nor* genes, we analyzed expression of *nor* genes in wild-type and *regR* mutant cells cultured anoxically or under 2% initial O_2_ in the absence or the presence of nitrate (Table 2). The presence of nitrate induced expression of a *norC-lacZ* transcriptional fusion in wild-type cells grown under anoxic conditions or 2% initial O_2_ about 14-fold and 9-fold, respectively. Interestingly, β-galactosidase activity derived from the *norC-lacZ* transcriptional fusion was about 38-fold lower in the *regR* mutant compared to wild-type levels in cells grown anoxically in BSN medium. However, *regR* mutation did not decrease *norC* expression compared to wild-type levels when cells were cultured in the same medium under 2% initial O_2_. By contrast, under the latter conditions levels of β-galactosidase activity were even higher in the *regR* mutant compared to the wild type, regardless of the presence or absence of nitrate. These results suggest that both nitrate and anoxic conditions are required for RegR-dependent induction of *nor* genes.

**Table 2:** β-Galactosidase activity derived from a *norC–lacZ* fusion present in *B. japonicum* wild type (2499), *regR* (2499RR) or *regS* (2499RS) mutant cells.

Strain	Relevant genotype	Miller units			
		Anoxia		2% O_2_	
		− nitrate	+ nitrate	− nitrate	+ nitrate
2499	wild type	76.2 (6.8)	1079 (70)	39.8 (11.3)	347.3 (52)
2499RR	*regR*	144 (41)	28.6 (3.9)	169.6 (25.7)	499.5 (22.6)
2499RS	*regS*	nd	1266 (90)	nd	nd

Cells were cultured under anoxic conditions or under 2% initial O_2_ in Bergersen minimal medium without (BS) or with nitrate (BSN). Data are means with standard error (in parenthesis) from at least three independent cultures, assayed in triplicate. nd, not determined.

Next, we studied the potential involvement of the sensor protein RegS on RegR-dependent induction of *nor* genes. As shown in Table 2, expression of *nor* genes in the *regS* mutant grown anoxically in BSN medium was very similar to that observed in wild-type cells, suggesting that RegR control of *norC* induction is independent on RegS. Likewise, only a weak influence of RegS on *norC* expression was observed in qRT-PCR analyses. While *norC* expression was 27-fold lower in the *regR* mutant compared to the wild-type strain, when cells were grown anoxically in BSN medium, only an about 3-fold decrease of *norC* expression relative to the wild type was observed in the *regS* mutant (data not shown).

**Figure 6:**
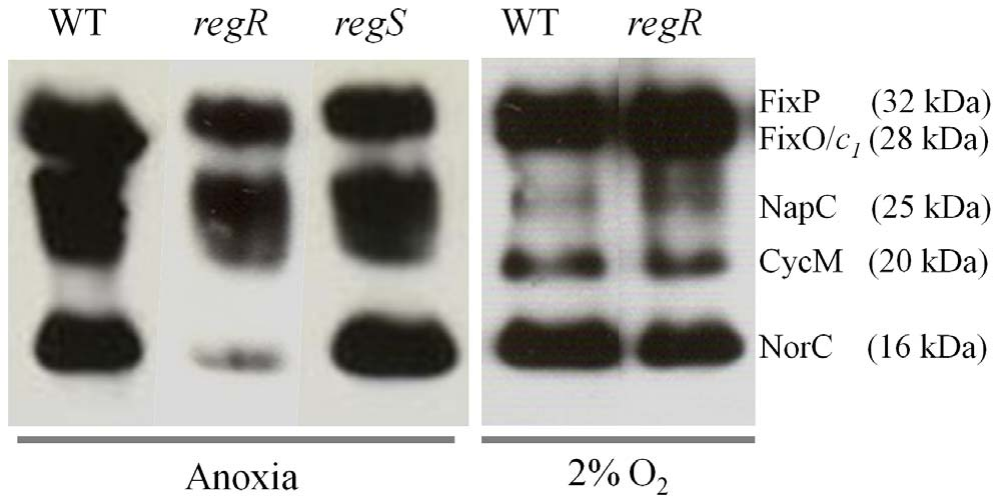
Heme-stained membrane proteins from cells of *B. japonicum* wild type (WT; lanes 1 and 4), *regR* mutant strain 2426 (lanes 2 and 5), and *regS* mutant strain 2409 (lane 3) cultured in BSN medium under anoxic conditions or 2% initial O_2_. About 20 µg membrane proteins were loaded per lane. Heme-stained *c*-type cytochromes identified previously are specified at the right margin along with their predicted mass. Note that all the samples were run in parallel on the same gel.

We also analyzed synthesis of NorC by heme *c* staining. In cells grown anoxically in BSN medium, NorC protein levels decreased in the *regR* mutant relative to the wild type (Fig. 6; lanes 1, 2). However, no difference of NorC expression was observed in *regS* mutant cells compared to wild-type cells (Fig. 6; lanes 1, 3). Furthermore, when cells were incubated under 2% initial O_2_ concentration in BSN medium, NorC expression did not decrease in the *regR* mutant (Fig. 6; lanes 4, 5).

Finally, we also tested the involvement of RegR in *nor* expression at the level of Nor activity by measuring NO consumption of wild-type and *regR* mutant cells cultured anoxically in BSN medium. As expected, NO consumption by *regR* mutant cells was drastically decreased (54±7 nmoles NO mg protein^−1^ min^−1^) compared to wild-type cells (832±7 nmoles NO mg protein^−1^ min^−1^).

## Discussion

The ability of *B. japonicum* to denitrify is well documented [4]. However, so far no information has been available about target genes controlled by RegR under denitrifying conditions. Our transcriptome analyses showed that expression of approximately 1,700 genes was altered in a *regR* mutant compared to the wild type when cells were grown anoxically in BSN medium. If only those genes are considered which showed a fold-change value of expression ≤−5 and ≥5 (343 genes; Table S3), the large majority of them (83%) are subject to positive control by RegR. This documents that this protein predominantly acts as an activator under the applied growth conditions, which extends the results obtained previously with *regR* mutant cells grown under oxic or microoxic free-living conditions, or as bacteroids in root nodules [25]. Notably, among this group of 343 genes we found the previously described RegR targets such as *fixR*, *nifA*, the blr1515-blr1516 operon that encodes a predicted multidrug efflux system [25], [47], and bll4130 encoding a LysR-type regulator, which validates the approach used in this work. Among the 620 genes anoxically induced whose expression differed in the *regR* mutant, a group of 344 genes were positively regulated (Figure 1). Based on this observation we propose a relevant role for RegR in *B. japonicum* denitrification. For example, we found among RegR targets structural denitrification genes (*nor*, *nos*) and genes participating in electron transport through the denitrification pathway (*cycA*, *cy*_2_), and the respective microarray data was validated by qRT-PCR. In this context, the involvement of *R. sphaeroides* PrrBA [48] and *Brucella suis* RegBA [49] in *nirK* regulation is worth to be mentioned. Likewise, insertional inactivation of the response regulatory *Agrobacterium tumefaciens actR* gene significantly reduced *nirK* expression as well as expression of *paz* encoding the electron-transport protein pseudoazurin [50]. Recently, it has been reported that the redox-responsive NtrYX and PrrBA two-component systems of *Brucella abortus* co-ordinately regulate the expression of denitrification in response to oxygen-limited conditions [51]–[53]. Results from our work indicate that, in contrast to *nor* and *nos* genes, *nirK* and *nap* denitrification genes were not under the control of RegR, which documents that *B. japonicum* denitrification genes differ with regard to their dependence on RegR. Notably, disparate regulation of *nap*, *nirK* and *nor* genes by FixK_2_ was found previously during transcription profiling studies with a *B. japonicum fixK*_2_ mutant strain grown under micro-oxic conditions [14]. Specifically, *nap*, *nirK*, and *nnrR*, but not *nor* or *nos* genes were among the targets of FixK_2_.

We have also identified and validated as RegR targets, *copCAB* genes encoding proteins involved in the assembly of periplasmic and secreted cuproproteins [54] which might be involved in maturation of the Cu-containing NirK or Nos enzymes. Furthermore, genes involved in NO detoxification and nitrate assimilation (blr2806-09) [44] as well as genes encoding transcriptional regulators were found to be controlled by RegR. Among the latter group was bll3466 encoding a FixK-like protein proposed to be involved in the negative feed-back of *fixK*_2_ expression [55] and bll4130 encoding a LysR-type regulator. Finally, *phyR* (bll7795) and the associated ECF σ-factor gene *ecfG* (blr7797) which contribute to stress tolerance and symbiotic proficiency of *B. japonicum* [56], were also identified as targets of RegR in this work.

Electrophoretic mobility shift experiments revealed RegR binding to the promoter regions of *norC*, *nosR*, bll3466, and bll4130. RegR binding to the latter, a LysR-type regulator gene, was also previously observed by Lindemann and co-workers (2007) [25]. Interestingly, we observed several bands of different mobilities in the case of the bll4130 promoter. It might be possible that at low concentration, RegR starts binding to one of the RegR-boxes found within this promoter region, and that at higher concentration it also binds to the secondary binding site of probably lower affinity. A hierarchical binding was also described for PhoB, the response regulator of the PhoRB two-component system which activates the transcription of several genes involved in phosphate uptake and assimilation [57]. By EMSA assays, these authors demonstrated that two PhoB dimers bind to two consecutive *pho* boxes in a hierarchical and cooperative manner. Using the FLOE technique we identified two transcriptional start sites in the *norC* promoter region (P1 and P2). P1 is the previously proposed FixK_2_-dependent start site [41], and P2 is a start site whose abundance was modulated by RegR under our experimental denitrifying conditions. Contrary to this work, previous primer extension analyses of *norC*, performed by using [γ-^32^P]ATP and the subsequent detection of extension products in denaturing polyacrylamide gels, only revealed the presence of the FixK_2_-dependent start site P1 but not the RegR-dependent start site P2 [41]. This apparent discrepancy could be due to the different growth conditions and methodological approaches used by Mesa and colleagues (2002) [41] and in this work. Anoxia and nitrate are required for RegR-dependent induction of *nor* genes. This is in line with microarray experiments performed previously to characterize the *B. japonicum* RegR regulon where *nor* genes were not among RegR-regulated genes in cells grown in PSY medium under microoxic conditions (max. 0.5% oxygen throughout cell cultivation) [25]. Those authors identified RegR-dependent genes in either free-living oxic or microoxic and symbiotic conditions and concluded that this protein contributes to redox regulation in *B. japonicum*. As shown here, RegR has also a regulatory role under anoxic conditions, and the presence of nitrate or a nitrogen oxide generated from nitrate reduction was crucial for RegR control of *nor* genes. Analogous to our findings, the presence of nitric oxide (NO) is required for ResDE-dependent anaerobic induction of *Bacillus subtillis nasDE* and *hmp* genes which encode a nitrite reductase and a NO-detoxifying flavohemoglobin, respectively. In this bacterium, NO inactivates the NO-sensitive NsrR transcriptional repressor of *nasDE* and *hmp* [58] leading to anaerobic induction of *nasDE* and *hmp* by ResDE. *B. japonicum* genome lacks genes coding for an obvious NsrR homolog [59], which, however, does not exclude that in addition to RegR other regulators contribute to the control of *B. japonicum nor* genes under denitrifying conditions.

RegSR two-component regulatory system comprises the membrane associated RegS histidine protein kinase and its cognate RegR response regulator [39]. The regulatory mechanism has been well-studied in the orthologous RegBA system of *R. capsulatus* [46], [60]. In this bacterium, the membrane-localized ubiquinone pool and the redox-active cysteine (Cys^265^) function as redox sensors that regulate RegB kinase activity which auto-phosphorylates and transfers the phosphoryl group to the RegA response regulator [61]–[64]. In this work, we made the intriguing observation that regulation of *norC* differed in *regS* and *regR* mutants. While deletion of *regR* abolished activation of *norC* under anoxic conditions in BSN medium, mutation of *regS* resulted in wild type-like expression *norC*. This is in line with previously described phenotypic differences between *regR* and *regS* mutants of *B. japonicum* [20], *regB* and *regA* mutants of *R. capsulatus* [65], or *roxS* and *roxR* mutants of *Pseudomonas aeruginosa* [66]. It might be possible that in the *B. japonicum regS* mutant, RegR is phosphorylated via cross-talk by an alternative sensor kinase. In fact, the two-component regulatory system encoded by *B. japonicum* genes blr1154 and blr1155 shows pronounced similaritiy to RegSR (H.M. Fischer, unpublished data), and thus is a candidate for the postulated cross-talk.

## Supporting Information

Table S1 **List of primers used for qRT-PCR experiments and EMSA assays.**
(DOCX)

Table S2 **Anoxically induced genes (as compared to oxic conditions) whose expression differed in the Δ** ***regR*** **strain relative to the wild type.**
(DOCX)

Table S3 **Differentially expressed genes by a factor of ≤−5 or ≥5 in the Δ** ***regR*** **strain grown anoxically and their putative operon members.**
(DOCX)
