# Supplementary material for: The Global Response Regulator RegR Controls Expression of Denitrification Genes in Bradyrhizobium japonicum
Source: PLoS One. 2014 Jun 20;9(6):e99011. doi: 10.1371/journal.pone.0099011 (PMC4064962; doi:10.1371/journal.pone.0099011)
Supplement: Table S1 — List of primers used for qRT-PCR experiments and EMSA assays. (DOCX) [file pone.0099011.s001.docx]

Table S1: List of primers used for qRT-PCR experiments and EMSA assays .

| Experimental technique | Gene | Forward primer | | Reverse primer | |  |
| --- | --- | --- | --- | --- | --- | --- |
| qRT-PCR | *sigA* | SigA-1069F^1^ | 5’-GAGATCATCGTCGAGGTGAAG-3’ | SigA-1155R^1^ | 5’-GCGCTTGTTGATGTCGTAGA-3’ | |
|  | *nosZ* | nosZ_for_1 | 5'-TCAGGTCACCGTCTACATCAC-3' | nosZ_rev_1 | 5'-CCATCTGGATACCGTAGTTCAC-3' | |
|  | *nosY* | nosY_for_1 | 5'-ATGACGCTGCTCCTGAGTTA-3' | nosY_rev_1 | 5'-CCGTAGCCGATCACTGTC-3' | |
|  | *norC* | norC_3_for | 5'-GCAGATGCCGCAGTTCAAC-3' | norC_3_rev | 5'-TGATCGTGCTCACCCATTG-3' | |
|  | *blr2808* | bll2808_1_for | 5'-TGGTCTCGACCAATCTGAAG-3' | blr2808_1_rev | 5'-CGATGACGAGCTTCTTGTA-3' | |
|  | *napE* | bsr7036_for_4 | 5'-GCCTTCCTGTTCCTGAC-3' | bsr7036_rev_4 | 5'-CCGGCAAACATCTGGTAGA-3' | |
|  | *napA* | bsr7038_for_1 | 5'-GAGCATCCGCTGCAGAAGA-3' | bsr7038_rev_1 | 5'-CGTGTACTCCGAGACGAACTTG-3' | |
|  | *cycA* | cycA_for_1 | 5'-AACAAGAATTCCGGCATCAC-3' | cycA_rev_1 | 5'-TGATCTCGGTCTCGTTCTTG-3' | |
|  | *copC* | bll2209_1_for | 5'-CAGGAATTCGTGGTCTTC-3' | bll2209_1_rev | 5'-TACGAGCCGTCGAACATC-3' | |
|  | *bll3466* | bll3466_for_1 | 5'-ACGAGCGATTCAAATCCAA-3' | bll3466_rev_1 | 5'-ACCGTCCGACAGGAGTTTA-3' | |
|  | *bll4130* | bll4130_for | 5'-ATATGGAGCGTCATGCCTTC-3' | bll4130_rev | 5'-TCTTGCGATAGGTTTTCTGGA-3' | |
|  | *cy_2_* | bll2388_for | 5'-GAATGTCATCGACCGCAAG-3' | bll2388_rev | 5'-TTGCATCAGAATAGGCGAAG-3' | |
|  |  |  |  |  |  | |
| EMSA | *bll2087* | 2087-23F^2^ | 5´-AGCAAGCTCTGGTGTCCAAG-3´ | 2087-24R^2^ | 5´-TAAACGTCAACGCGACAAAG-3´ | |
|  | *bll2087* | 2087-11F^3^ | 5´-TACGCTGCCTACACCCAAT-3´ | 2087-12R^3^ | 5´-AGGAGGTAATGCCGTCTTGT-3´ | |
|  | *norC* | EMSA_NorC_F | 5'-GTCATCGTCGTGCTGTTTG-3' | EMSA_NorC_R | 5'-GAGCCGCCGTAGAAGACG-3' | |
|  | *nosR* | EMSA_NosR_2F | 5'-CGCTCATCAGCAGCGAAG-3' | EMSA_NosR_R | 5'-CCGCTTGGGTTAGAAAATCC-3' | |
|  | *bll3466* | EMSA_3466_F | 5'-CACCGACCTGTCCCTTGGTAC-3' | EMSA_3466_R | 5'-CGAGGTCTTTGAGCGAATTG-3' | |
|  | *bll4130* | EMSA_4130_F | 5'-CGTCGAAATCATGCCTTGC-3' | EMSA_4130_R | 5'-CGTCCAGGGCTTCTTCAC-3' | |
|  | *cy_2_* | EMSA_2388_F | 5'-CGGTTGATGCAGGACAAAG-3' | EMSA_2388_R | 5'-GCATGAGCACGAAGATCAGA-3' | |
|  | *blr2806* | EMSA_2806_F | 5'-GCAATTCGAACAAGCCACTG-3' | EMSA_2806_R | 5'-GCCAGTCTGAAATCCAGGTC-3' | |

^1^ Oligonucleotides for amplification of the primary sigma factor *sigA* gene used previously for normalization in qRT-PCR experiments (Lindemann et al., 2007).

^2^ Oligonucleotides for amplification of a previously demonstrated region binding by RegR (Hauser et al., 2006), used as positive control in EMSA assays.

^3^ Oligonucleotides for amplification a DNA region not binding by RegR (Hauser et al., 2006), used as negative control in EMSA assays.
