# Supplementary material for: The Global Response Regulator RegR Controls Expression of Denitrification Genes in Bradyrhizobium japonicum
Source: PLoS One. 2014 Jun 20;9(6):e99011. doi: 10.1371/journal.pone.0099011 (PMC4064962; doi:10.1371/journal.pone.0099011)
Supplement: Table S2 — Anoxically induced genes (as compared to oxic conditions) whose expression differed in the Δ regR strain relative to the wild type. (DOCX) [file pone.0099011.s002.docx]

Table S2: Anoxically induced genes (as compared to oxic conditions) whose expression differed in the Δ*regR* strain relative to the wild type^a^.

| **Class and gene no.^b^** | **Putative operon member (gene no.^)c^** | | **Gene name^d^** | **Description^e^** | **Relative change in expression (*n*-fold)^f^** | | | | | | |
| --- | --- | --- | --- | --- | --- | --- | --- | --- | --- | --- | --- |
|  |  | |  |  | **WT_anoxic_vs_WT_oxic^g^** | | | | | | **Δ*regR*_anoxic_vs_WT_anoxic^h^** |
| **Class 1 (downregulated in the Δ*regR* strain)** | | | |  |  | | | | | |  |
| *bll0100* |  | |  | ferredoxin NADP+ reductase | 2.0 | | | | | | -4.4 |
| *bsr0136* |  | |  | hypothetical protein | 2.2 | | | | | | -2.8 |
| *bll0161* |  | |  | hypothetical protein | 3.0 | | | | | | -2.2 |
| *blr0274* |  | |  | hypothetical protein | 11.1 | | | | | | -12.7 |
| *bll0301* |  | | *ragC* | cation efflux protein | 2.4 | | | | | | -3.2 |
|  | *bll0300* | | *ragD* | RagD protein | 2.4 | | | | | | -4.2 |
| *blr0305* |  | |  | hypothetical protein | 8.9 | | | | | | -9.5 |
| *blr0306* |  | |  | hypothetical protein | 6.4 | | | | | | -4.1 |
| *blr0314* |  | | *nosR* | nitrous oxide reductase expression regulator | 122.8 | | | | | | -2.2 |
|  | *blr0315* | | *nosZ* | nitrous-oxide reductase precursor | 95.5 | | | | | | -7.6 |
|  | *blr0316* | | *nosD* | periplasmic copper-binding precursor | 38.7 | | | | | | -12.1 |
|  | *blr0317* | | *nosF* | copper ABC transporter | 37.1 | | | | | | -14.2 |
|  | *blr0318* | | *nosY* | nitrous oxide metabolic protein | 76.7 | | | | | | -10.5 |
|  | *blr0319* | | *nosL* | NosL protein | 39.1 | | | | | | -11.1 |
|  | *blr0320* | | *nosX* | NosX protein | 29.4 | | | | | | -10.5 |
| *bll0322* |  | | *otsA* | probable trehalose-6-phosphate synthase | 6.4 | | | | | | -5.5 |
| *bll0342* |  | | *fah* | fumarylacetoacetase | 3.9 | | | | | | -8.6 |
| *bll0346* |  | |  | hypothetical protein | − | | | | | | − |
|  | *bsl0345* | |  | hypothetical protein | − | | | | | | − |
|  | *bll0344* | |  | hypothetical protein | − | | | | | | − |
|  | *bll0343* | |  | homogentisate 1.2-dioxygenase | 7.0 | | | | | | -9.6 |
| *blr0401* |  | |  | hypothetical protein | 14.4 | | | | | | -5.6 |
| *bsr0421* |  | | *rpmA* | 50S ribosomal protein L27 | 2.2 | | | | | | -4.6 |
| *blr0444* |  | |  | hypothetical protein | 4.5 | | | | | | -7.5 |
| *bll0464* |  | |  | hypothetical protein | 4.2 | | | | | | -2.1 |
| *bll0465* |  | |  | hypothetical protein | 2.7 | | | | | | -10.6 |
| *blr0468* |  | | *ccmB* | heme exporter protein B | − | | | | | | − |
|  | *blr0469* | | *ccmC* | heme exporter protein C | − | | | | | | − |
|  | *bsr0470* | | *ccmD* | heme exporter protein D | − | | | | | | − |
|  | *blr0471* | | *ccmG* | thiol:disulfide interchange protein | 3.9 | | | | | | -2.2 |
| *bll0506* |  | |  | hypothetical protein | 5.0 | | | | | | -4.8 |
|  | *bll0505* | |  | hypothetical protein | 3.3 | | | | | | -5.4 |
| *bll0527* |  | |  | hypothetical oxidoreductase | 2.1 | | | | | | -3.0 |
| *blr0536* |  | |  | transcriptional regulatory protein | 9.1 | | | | | | -2.5 |
| *bll0556* |  | |  | hypothetical protein | 2.1 | | | | | | -2.5 |
| *blr0806* |  | |  | hypothetical protein | 3.5 | | | | | | -7.6 |
| *blr0807* |  | |  | succinate-semialdehyde dehydrogenase | 7.3 | | | | | | -2.2 |
| *bll0818* |  | |  | hypothetical protein | 106.8 | | | | | | -20.7 |
| *bsr0858* |  | |  | hypothetical protein | 9.7 | | | | | | -11.8 |
| *bsr0859* |  | |  | hypothetical protein | 4.5 | | | | | | -8.0 |
| *bsr0862* |  | |  | hypothetical protein | 7.9 | | | | | | -2.6 |
| *bll0888* |  | |  | hypothetical protein | 5.0 | | | | | | -3.8 |
| *bll0905* |  | | *regS* | two-component sensor histidine kinase | − | | | | | | − |
|  | *bll0904* | | *regR* | two-component response regulator | 5.3 | | | | | | -91.7 |
| *blr0908* |  | |  | hypothetical protein | 2.0 | | | | | | -2.4 |
| *bsl0950* |  | |  | hypothetical protein | 13.5 | | | | | | -5.3 |
| *blr1091* |  | | *pstS* | ABC transporter phosphate-binding protein | 3.3 | | | | | | -2.1 |
| *bll1101* |  | | *apaG* | hypothetical protein | 3.0 | | | | | | -2.2 |
| *bsl1208* |  | |  | hypothetical protein | 3.8 | | | | | | -3.2 |
| *bll1231* |  | |  | hypothetical protein | − | | | | | | − |
|  | *bll1230* | |  | 3-oxoacyl-[acyl-carrier-protein] reductase | 7.7 | | | | | | -4.1 |
| *blr1263* |  | |  | hypothetical protein | − | | | | | | − |
|  | *blr1264* | |  | hypothetical protein | 4.0 | | | | | | -6.4 |
|  | *blr1265* | |  | hypothetical protein | 4.7 | | | | | | -6.3 |
| *bll1285* |  | |  | hypothetical protein | 6.3 | | | | | | -151.1 |
| *blr1289* |  | |  | hypothetical protein | 174.1 | | | | | | -8.1 |
| *bll1299* |  | |  | hypothetical protein | 2.2 | | | | | | -3.5 |
| *blr1311* |  | |  | outer membrane protein | 7.2 | | | | | | -2.5 |
| *bsl1312* |  | |  | hypothetical protein | 5.5 | | | | | | -3.3 |
| *bsl1363* |  | |  | hypothetical protein | 14.5 | | | | | | -4.6 |
| *blr1429* |  | |  | hypothetical protein | 4.0 | | | | | | -8.1 |
| *bll1465* |  | |  | hypothetical protein | 4.1 | | | | | | -4.1 |
| *bll1467* |  | |  | hypothetical protein | 10.4 | | | | | | -5.5 |
|  | *bll1466* | |  | hypothetical protein | 12.5 | | | | | | -11.9 |
| *blr1468* |  | |  | hypothetical protein | 35.4 | | | | | | -26.1 |
|  | *blr1469* | |  | hypothetical protein | 35.6 | | | | | | -22.3 |
| *bsr1472* |  | |  | hypothetical protein | 7.3 | | | | | | -2.1 |
| *bsl1473* |  | |  | hypothetical protein | 62.0 | | | | | | -14.9 |
| *blr1482* |  | |  | ABC transporter sulfate-binding protein | 11.1 | | | | | | -2.7 |
| *blr1483* |  | |  | sulfate ABC transporter permease protein | 5.7 | | | | | | -2.8 |
|  | *blr1484* | |  | sulfate ABC transporter permease protein | 3.9 | | | | | | -3.1 |
|  | *blr1485* | |  | sulfate ABC transporter ATP-binding protein | 3.8 | | | | | | -4.4 |
|  | *blr1486* | |  | hypothetical protein | − | | | | | | − |
| *bll1523* |  | | *gapA* | glyceraldehyde-3-phosphate dehydrogenase | 2.3 | | | | | | -2.9 |
| *blr1601* |  | |  | ABC transporter substrate-binding protein | 2.1 | | | | | | -2.7 |
|  | *blr1602* | |  | ABC transporter permease protein | − | | | | | | − |
|  | *blr1603* | |  | ABC transporter permease protein | − | | | | | | − |
|  | *blr1604* | |  | ABC transporter ATP-binding protein | − | | | | | | − |
| *blr1617* |  | | *trbL* | conjugal transfer protein | − | | | | | | − |
|  | *blr1618* | | *trbF* | probable conjugal transfer protein | − | | | | | | − |
|  | *blr1619* | | *trbG* | conjugal transfer protein | − | | | | | | − |
|  | *blr1620* | | *trbI* | conjugal transfer protein | 2.4 | | | | | | -4.5 |
|  | *bsr1621* | |  | hypothetical protein | 2.8 | | | | | | -12.5 |
| *bll1766* |  | |  | outer membrane protein | 8.0 | | | | | | -10.2 |
| *bll2007* |  | | *hemN_1_* | coproporphyrinogen III oxidase | 72.4 | | | | | | -3.7 |
| *bsl2064* |  | |  | hypothetical protein bsl2064 | 2.1 | | | | | | -5.3 |
| *bll2067* |  | | *nfeC* | nodulate formation efficiency C protein | 4.1 | | | | | | -2.9 |
| *blr2177* |  | |  | hypothetical protein | 2.0 | | | | | | -2.7 |
|  | *blr2178* | |  | two-component hybrid sensor and regulator | − | | | | | | − |
| *bsl2212* |  | |  | hypothetical protein | 27.1 | | | | | | -6.8 |
|  | *bll2211* | | *copB* | copper tolerance protein | 16.7 | | | | | | -15.2 |
|  | *bll2210* | | *copA* | multicopper oxidase | 9.8 | | | | | | -10.7 |
|  | *bll2209* | | *copC* | copper tolerance protein | 6.6 | | | | | | -16.1 |
|  | *bll2208* | |  | hypothetical protein | 8.2 | | | | | | -9.2 |
| *bll2213* |  | |  | hypothetical protein | 9.2 | | | | | | -4.8 |
| *bll2388* |  | | *cy_2_* | cytochrome *c_2_* | 232.9 | | | | | | -8.3 |
| *bsl2407* |  | |  | hypothetical protein | 14.4 | | | | | | -7.8 |
| *blr2426* |  | |  | hypothetical protein | − | | | | | | − |
|  | *blr2427* | |  | acetyl-CoA acetyltransferase | − | | | | | | − |
|  | *blr2428* | |  | putative fatty acid oxidation complex alpha subunit | − | | | | | | − |
|  | *blr2429* | |  | hypothetical protein | 3.1 | | | | | | -2.2 |
| *bll2445* |  | |  | oxidoreductase | 5.1 | | | | | | -3.2 |
| *bll2449* |  | |  | hypothetical protein | − | | | | | | − |
|  | *bll2448* | |  | probable cellulose synthase catalytic subunit | 5.5 | | | | | | -3.6 |
| *blr2451* |  | |  | hypothetical protein | 12.0 | | | | | | -2.2 |
| *bll2462* |  | |  | hypothetical protein | 4.5 | | | | | | -6.0 |
| *bll2465* |  | |  | MoxR family protein | 10.6 | | | | | | -7.5 |
|  | *bll2464* | |  | hypothetical protein | 5.0 | | | | | | -4.6 |
|  | *bll2463* | |  | hypothetical protein | 6.3 | | | | | | -6.6 |
| *blr2487* |  | |  | hypothetical protein | 3.3 | | | | | | -2.5 |
| *blr2501* |  | |  | hypothetical protein | 4.3 | | | | | | -15.1 |
| *blr2505* |  | |  | hypothetical protein | 10.7 | | | | | | -11.0 |
| *blr2511* |  | | *moeB* | molybdopterin biosynthesis protein B | 2.7 | | | | | | -2.5 |
| *blr2557* |  | |  | RNA polymerase sigma-70 factor | 5.9 | | | | | | -2.1 |
| *bsl2596* |  | |  | hypothetical protein | 22.6 | | | | | | -7.8 |
| *bsl2602* |  | |  | hypothetical protein | 5.3 | | | | | | -9.6 |
| *blr2603* |  | |  | hypothetical protein | 10.5 | | | | | | -3.4 |
| *blr2668* |  | |  | hypothetical protein | 15.0 | | | | | | -2.2 |
| *blr2694* |  | |  | VirG-like two component response regulator | 5.3 | | | | | | -12.4 |
| *bll2734* |  | |  | sulfur oxidation protein SoxY | − | | | | | | − |
|  | *bll2733* | |  | probable sulfur oxidation protein | 2.6 | | | | | | -2.5 |
|  | *bll2732* | |  | putative cytochrome *c* | − | | | | | | − |
|  | *bll2731* | |  | probable ABC transporter substrate-binding protein | − | | | | | | − |
|  | *bll2730* | |  | probable ABC transporter permease protein | − | | | | | | − |
|  | *bll2729* | |  | probable ABC transporter permease protein | − | | | | | | − |
| *bll2737* |  | |  | oxidoreductase with iron-sulfur subunit | 12.8 | | | | | | -14.2 |
|  | *bll2736* | |  | putative aldehyde dehydrogenase protein | 7.0 | | | | | | -10.3 |
| *bll2743* |  | |  | hypothetical protein | 2.8 | | | | | | -3.6 |
| *bll2752* |  | |  | probable glycosyl transferase | 12.5 | | | | | | -4.1 |
| *blr2753* |  | |  | ABC transporter HlyB/MsbA family | 24.9 | | | | | | -4.5 |
| *blr2806^i^* |  | |  | nitrite extrusion protein | 27.8 | | | | | | -10.3 |
|  | *blr2807* | | *bjgb* | probable bacterial hemoglobin | 14.9 | | | | | | -4.0 |
|  | *blr2808* | |  | putative FAD and NAD(P)H-binding reductase protein | 21.4 | | | | | | -9.2 |
|  | *blr2809* | | *nasA* | nitrate reductase large subunit | 5.0 | | | | | | -5.5 |
| *bll2830* |  | |  | probable enoyl-CoA hydratase | 2.3 | | | | | | -3.5 |
| *bll2850* |  | |  | probable 6-phosphofructokinase | − | | | | | | − |
|  | *bll2849* | |  | hypothetical protein | 14.3 | | | | | | -8.4 |
| *bll2851* |  | |  | hypothetical protein | 3.0 | | | | | | -2.5 |
| *blr2852* |  | |  | hypothetical protein | 19.4 | | | | | | -2.1 |
| *blr2932* |  | |  | hypothetical protein | 2.7 | | | | | | -3.7 |
| *blr2943* |  | |  | enoyl-CoA hydratase | − | | | | | | − |
|  | *blr2944* | |  | hypothetical protein | 2.3 | | | | | | -2.7 |
|  | *blr2945* | |  | hypothetical protein | − | | | | | | − |
| *blr2983* |  | |  | hypothetical oxidodeductase | 2.2 | | | | | | -4.2 |
| *blr3017* |  | |  | hypothetical protein | 4.0 | | | | | | -2.1 |
| *bll3037* |  | |  | hypothetical protein | 4.8 | | | | | | -11.9 |
| *bll3087* |  | |  | transcriptional regulatory protein | 3.8 | | | | | | -2.2 |
|  | *bll3086* | |  | putative arsenate reductase | − | | | | | | − |
|  | *bll3085* | |  | sodium bile acid symporter family protein | − | | | | | | − |
| *blr3130* |  | |  | serine protease DO-like precursor | 2.3 | | | | | | -2.9 |
| *blr3169* |  | |  | hypothetical protein | 10.8 | | | | | | -38.3 |
| *blr3212* |  | | *norE* | nitric oxide reductase subunit E | 403.6 | | | | | | -13.6 |
|  | *bsr3213* | |  | hypothetical protein | 46.0 | | | | | | -10.8 |
| *blr3214* |  | | *norC* | nitric oxide reductase subunit C | 340.9 | | | | | | -5.2 |
|  | *blr3215* | | *norB* | nitric oxide reductase subunit B | 291.4 | | | | | | -4.9 |
|  | *blr3216* | | *norQ* | NorQ protein | 335.1 | | | | | | -7.2 |
|  | *blr3217* | | *norD* | NorD protein | 65.8 | | | | | | -11.3 |
| *blr3218* |  | |  | putative hydrolase phosphatase protein | 25.7 | | | | | | -5.8 |
|  | *blr3219* | |  | probable transcriptional regulator | 9.7 | | | | | | -4.5 |
| *blr3456* |  | |  | hypothetical protein | − | | | | | | − |
|  | *blr3457* | | *pta* | phosphate acetyltransferase | − | | | | | | − |
|  | *blr3458* | | *ackA2* | acetate/propionate kinase | − | | | | | | − |
|  | *blr3459* | | *fabI* | enoyl-(acyl carrier protein) reductase | 8.4 | | | | | | -2.3 |
| *bll3466* |  | | *fixK* | transcriptional regulator FixK | 8.0 | | | | | | -5.2 |
| *bll3594* |  | |  | hypothetical protein | 3.1 | | | | | | -4.1 |
|  | *bll3593* | |  | hypothetical protein | 3.0 | | | | | | -2.7 |
| *blr3607* |  | |  | hypothetical protein | 2.3 | | | | | | -4.6 |
| *bll3611* |  | |  | hypothetical protein | 4.5 | | | | | | -2.5 |
| *bll3717* |  | | *lipA* | lipoyl synthase | 2.3 | | | | | | -5.8 |
| *bll3765* |  | |  | glutamine amidotransferase | 2.2 | | | | | | -6.9 |
|  | *bll3764* | |  | hypothetical protein | 12.3 | | | | | | -6.1 |
| *blr3767* |  | |  | hypothetical protein | 4.0 | | | | | | -2.1 |
| *bll3768* |  | |  | hypothetical protein | 13.2 | | | | | | -6.6 |
| *blr3769* |  | |  | hypothetical protein | 6.0 | | | | | | -7.7 |
|  | *blr3770* | |  | hypothetical protein | 6.4 | | | | | | -38.0 |
| *blr3860* |  | |  | hypothetical protein | 17.4 | | | | | | -21.5 |
| *bsl3938* |  | |  | putative biotinylated protein | 9.2 | | | | | | -10.6 |
| *bsl4014* |  | |  | hypothetical protein | 12.1 | | | | | | -5.6 |
| *bll4065* |  | |  | hypothetical protein | 3.1 | | | | | | -2.2 |
| *bsr4099* |  | |  | hypothetical protein | − | | | | | | − |
|  | *blr4100* | |  | hypothetical protein | 7.2 | | | | | | -16.4 |
| *bll4149* |  | |  | putative glutathione peroxidase | 8.2 | | | | | | -4.1 |
| *bll4166* |  | |  | hypothetical protein | 3.3 | | | | | | -2.2 |
| *bll4168* |  | |  | hypothetical protein | − | | | | | | − |
|  | *bsl4167* | |  | putative glutamine synthetase translation inhibitor | 8.9 | | | | | | -6.8 |
| *bsr4179* |  | |  | hypothetical protein | 2.3 | | | | | | -2.8 |
| *blr4182* |  | |  | hypothetical protein | 3.0 | | | | | | -28.2 |
| *bll4218* |  | |  | hypothetical protein | 3.9 | | | | | | -26.0 |
| *blr4219* |  | |  | hypothetical protein | 4.2 | | | | | | -4.1 |
| *bll4234* |  | |  | hypothetical protein | 5.4 | | | | | | -2.8 |
| *blr4238* |  | |  | hypothetical protein | 2.5 | | | | | | -3.1 |
| *bll4247* |  | |  | hypothetical protein | 3.5 | | | | | | -5.2 |
| *bll4278* |  | |  | hypothetical protein | 4.1 | | | | | | -2.9 |
| *bsl4407* |  | |  | hypothetical protein | 7.0 | | | | | | -2.8 |
| *bsr4408* |  | |  | hypothetical protein | 13.1 | | | | | | -9.5 |
| *bll4412* |  | |  | hypothetical protein | 10.8 | | | | | | -7.6 |
| *bsl4437* |  | |  | hypothetical protein | 7.7 | | | | | | -10.1 |
| *blr4463* |  | |  | probable ABC transporter substrate-binding protein | 2.7 | | | | | | -2.3 |
|  | *blr4464* | |  | probable ABC transporter ATP-binding/permease protein | | | | − | | | − |
| *blr4465* |  | |  | hypothetical protein | 3.0 | | | | | | -2.1 |
| *bsr4491* |  | |  | RNA-binding protein Hfq | 2.0 | | | | | | -2.5 |
| *blr4588* |  | |  | hypothetical protein | 2.6 | | | | | | -2.9 |
| *bsl4622* |  | |  | hypothetical protein | 3.6 | | | | | | -4.9 |
| *bsl4623* |  | |  | hypothetical protein | 15.6 | | | | | | -4.8 |
| *bll4722* |  | |  | hypothetical protein | 2.9 | | | | | | -3.2 |
| *blr4723* |  | |  | hypothetical protein | 6.2 | | | | | | -5.5 |
| *bll4741* |  | |  | putative arylsulfatase protein | 2.5 | | | | | | -2.7 |
| *blr4795* |  | |  | putative hydrolase | 14.9 | | | | | | -2.9 |
| *bll4828* |  | |  | hypothetical protein bll4828 | 31.7 | | | | | | -3.1 |
| *blr4870* |  | |  | MFS permease | 4.7 | | | | | | -2.6 |
| *bll4880* |  | |  | hypothetical protein | 2.2 | | | | | | -3.0 |
|  | *bll4879* | |  | hypothetical protein | 2.8 | | | | | | -2.9 |
|  | *bll4878* | |  | possible Copper export protein | − | | | | | | − |
| *blr4891* |  | |  | hypothetical protein | 4.6 | | | | | | -3.8 |
| *bll4896* |  | |  | ABC transporter substrate-binding protein | 2.1 | | | | | | -5.7 |
| *bll4919* |  | | *nuoA* | NADH dehydrogenase alpha subunit | 2.3 | | | | | | -2.2 |
|  | *bll4918* | | *nuoB* | NADH dehydrogenase beta subunit | − | | | | | | − |
|  | *bll4917* | | *nuoC* | NADH dehydrogenase subunit C | − | | | | | | − |
|  | *bll4916* | | *nouD* | NADH dehydrogenase delta subunit | − | | | | | | − |
|  | *bll4915* | |  | hypothetical protein | − | | | | | | − |
|  | *bll4914* | |  | ATP synthase subunit E | − | | | | | | − |
|  | *bsl4913* | |  | hypothetical protein | − | | | | | | − |
|  | *bll4912* | | *nuoF* | NADH ubiquinone oxidoreductase chain F | − | | | | | | − |
|  | *bll4911* | | *nuoG* | NADH dehydrogenase gamma subunit | − | | | | | | − |
|  | *bll4910* | | *nuoH* | NADH dehydrogenase subunit H | − | | | | | | − |
|  | *bll4909* | | *nuoI* | NADH dehydrogenase subunit I | − | | | | | | − |
| *blr4930* |  | |  | hypothetical protein | 26.9 | | | | | | -3.4 |
|  | *blr4931* | |  | hypothetical protein | 71.4 | | | | | | -13.4 |
|  | *blr4932* | |  | putative cation efflux system protein | 62.1 | | | | | | -22.0 |
| *blr4933* |  | |  | probable cation efflux system protein | 37.0 | | | | | | -26.5 |
| *bll4983* |  | |  | hypothetical protein bll4983 | 2.8 | | | | | | -6.9 |
| *blr4984* |  | |  | transcriptional regulatory protein | 2.1 | | | | | | -6.8 |
| *bll4985* |  | |  | hypothetical protein | 7.0 | | | | | | -11.4 |
| *bsl5034* |  | |  | hypothetical protein | 3.1 | | | | | | -5.1 |
| *bsl5035* |  | |  | hypothetical protein | 7.9 | | | | | | -4.6 |
| *bll5043* |  | |  | hypothetical protein | − | | | | | | − |
|  | *bll5042* | |  | hypothetical protein | 6.5 | | | | | | -3.1 |
|  | *bll5041* | |  | hypothetical protein | 4.0 | | | | | | -4.8 |
|  | *bll5040* | |  | hypothetical protein | 2.0 | | | | | | -2.9 |
| *bll5081* |  | |  | putative multidrug resistance protein | − | | | | | | − |
|  | *bll5080* | |  | AcrB/AcrD/AcrF family protein | 9.3 | | | | | | -2.7 |
|  | *bll5079* | |  | hypothetical protein | 10.3 | | | | | | -5.6 |
| *bll5130* |  | |  | hypothetical protein | 2.2 | | | | | | -2.2 |
|  | *bll5129* | |  | NTP pyrophosphohydrolase MutT family | − | | | | | | − |
| *bll5205* |  | |  | hypothetical protein | 3.0 | | | | | | -2.9 |
| *bsl5208* |  | |  | hypothetical protein | − | | | | | | − |
|  | *bll5207* | |  | hypothetical protein | 8.2 | | | | | | -4.9 |
| *blr5292* |  | |  | hypothetical protein | 4.4 | | | | | | -7.6 |
| *bsl5321* |  | |  | hypothetical protein | 2.3 | | | | | | -3.3 |
|  | *bll5320* | |  | hypothetical protein | − | | | | | | − |
| *bll5323* |  | |  | hypothetical protein | 9.8 | | | | | | -2.8 |
| *bll5324* |  | |  | hypothetical protein | 10.0 | | | | | | -5.3 |
| *blr5341* |  | |  | hypothetical protein | 17.2 | | | | | | -5.4 |
| *bll5373* |  | |  | probable short-chain dehydrogenase | 2.3 | | | | | | -7.1 |
|  | *bll5372* | |  | hypothetical protein | − | | | | | | − |
| *blr5441* |  | |  | hypothetical protein | 4.1 | | | | | | -3.8 |
| *bll5475* |  | |  | putative formate dehydrogenase | 13.4 | | | | | | -6.4 |
| *bll5477* |  | |  | similar to formate dehydrogenase | 12.6 | | | | | | -8.4 |
|  | *bll5476* | |  | formate dehydrogenase iron-sulfur subunit | 22.0 | | | | | | -7.6 |
| *bll5480* |  | |  | putative chaperone | 9.7 | | | | | | -3.1 |
|  | *bsl5479* | |  | hypothetical protein | 12.6 | | | | | | -3.3 |
|  | *bll5478* | |  | similar to formate dehydrogenase | 14.5 | | | | | | -3.5 |
| *bll5481* |  | |  | hypothetical protein | 2.3 | | | | | | -2.0 |
| *blr5502* |  | |  | hypothetical protein | 10.5 | | | | | | -10.5 |
| *bll5510* |  | |  | outer-membrane immunogenic protein precursor | 7.2 | | | | | | -4.5 |
| *blr5512* |  | |  | hypothetical protein | 2.5 | | | | | | -4.6 |
| *blr5554* |  | |  | hypothetical protein | 7.0 | | | | | | -3.4 |
| *bll5555* |  | |  | hypothetical protein | 37.3 | | | | | | -13.2 |
| *blr5556* |  | |  | hypothetical protein | 4.6 | | | | | | -7.7 |
| *bll5570* |  | |  | hypothetical protein | 24.0 | | | | | | -3.0 |
| *bsr5571* |  | |  | hypothetical protein | 18.9 | | | | | | -2.7 |
| *bll5579* |  | |  | hypothetical protein | 2.0 | | | | | | -2.8 |
| *blr5597* |  | |  | carboxypeptidase | 2.0 | | | | | | -2.2 |
| *bll5643* |  | |  | hypothetical protein | 3.1 | | | | | | -2.3 |
| *bsr5670* |  | |  | hypothetical protein | 8.9 | | | | | | -8.1 |
| *blr5675* |  | |  | ABC transporter substrate-binding protein | 2.1 | | | | | | -3.9 |
| *blr5693* |  | |  | probable substrate-binding protein | 8.9 | | | | | | -7.7 |
| *bsl5717* |  | |  | hypothetical protein | 4.2 | | | | | | -4.8 |
| *bsr5760* |  | |  | hypothetical protein | 18.3 | | | | | | -4.5 |
| *bll5807* |  | |  | hypothetical protein | 6.4 | | | | | | -4.9 |
| *bll5866* |  | |  | hypothetical protein | 11.0 | | | | | | -4.4 |
| *bll5899* |  | |  | hypothetical protein | 3.0 | | | | | | -4.0 |
| *blr5909* |  | |  | hypothetical protein | 2.1 | | | | | | -4.1 |
| *bll6012* |  | |  | hypothetical protein | 2.4 | | | | | | -2.2 |
| *blr6059* |  | |  | putative cyclase | 4.6 | | | | | | -3.7 |
| *bll6065* |  | |  | ABC transporter permease protein | − | | | | | | − |
|  | *bll6064* | |  | ABC transporter ATP-binding protein | − | | | | | | − |
|  | *bll6063* | |  | ABC transporter substrate-binding protein | 9.2 | | | | | | -2.2 |
| *bll6121* |  | |  | probable sulfite oxidase | 3.7 | | | | | | -4.5 |
|  | *bll6120* | |  | putative sulfite oxidase cytochrome subunit | 3.4 | | | | | | -4.7 |
| *blr6123* |  | |  | hypothetical protein | 42.0 | | | | | | -3.7 |
| *blr6167* |  | |  | hypothetical protein | 2.8 | | | | | | -5.6 |
| *bll6168* |  | |  | hypothetical protein | 3.4 | | | | | | -4.3 |
| *bsr6217* |  | |  | hypothetical protein | 3.2 | | | | | | -3.6 |
|  | *blr6218* | |  | putative oxidoreductase protein | − | | | | | | − |
|  | *blr6219* | |  | putative aldehyde dehydrogenase | − | | | | | | − |
| *bll6221* |  | |  | Rieske iron-sulfur protein | 3.0 | | | | | | -2.4 |
| *bll6222* |  | |  | probable Sec-independent protein translocase protein | 44.5 | | | | | | -3.0 |
| *bll6223* |  | | *hbdA* | 3-hydroxybutyryl-CoA dehydrogenase | 2.1 | | | | | | -2.2 |
| *bll6252* |  | |  | transcriptional regulatory protein | 4.8 | | | | | | -2.6 |
| *bll6262* |  | | *osmC* | probable osmotically inducible protein | 2.6 | | | | | | -5.4 |
|  | *bll6261* | |  | hypothetical protein | 2.3 | | | | | | -5.7 |
|  | *bll6260* | |  | methionine sulfoxide reductase A | − | | | | | | − |
| *blr6269* |  | |  | hypothetical protein | 2.1 | | | | | | -2.3 |
| *bll6449* |  | |  | hypothetical protein | 19.3 | | | | | | -4.7 |
| *bll6455* |  | |  | ABC transporter substrate-binding protein | 195.0 | | | | | | -2.4 |
|  | *bll6454* | |  | ABC transporter permease protein | 111.9 | | | | | | -2.8 |
|  | *bll6453* | |  | ABC transporter ATP-binding protein | 53.1 | | | | | | -3.5 |
|  | *bll6452* | | *acd* | acyl-CoA dehydrogenase | 123.0 | | | | | | -5.8 |
|  | *bll6451* | |  | probable alkanesulfonate monooxygenase | 36.8 | | | | | | -9.5 |
|  | *bll6450* | |  | probable substrate-binding protein | 23.3 | | | | | | -10.2 |
| *bll6468* |  | |  | hypothetical protein | 2.2 | | | | | | -2.5 |
| *blr6472* |  | |  | hypothetical protein | 8.7 | | | | | | -2.7 |
| *bll6525* |  | |  | hypothetical protein | 8.5 | | | | | | -3.1 |
| *bll6527* |  | |  | hypothetical protein | 2.7 | | | | | | -3.2 |
| *bll6529* |  | |  | hypothetical protein | 5.4 | | | | | | -4.3 |
|  | *bsl6528* | |  | hypothetical protein | − | | | | | | − |
| *bll6540* |  | |  | putative oxidoreductase | 15.5 | | | | | | -2.3 |
| *bsl6560* |  | |  | hypothetical protein | 5.1 | | | | | | -2.5 |
| *blr6563* |  | |  | hypothetical protein | 2.6 | | | | | | -8.3 |
|  | *blr6564* | |  | putative dihydroflavonol-4-reductase | − | | | | | | − |
|  | *blr6565* | |  | hypothetical protein | − | | | | | | − |
| *blr6582* |  | |  | hypothetical protein | 3.5 | | | | | | -3.3 |
| *bsl6653* |  | |  | hypothetical protein | 4.0 | | | | | | -4.4 |
| *blr6667* |  | |  | hypothetical protein | 4.2 | | | | | | -2.2 |
| *bsr6700* |  | |  | hypothetical protein | 5.2 | | | | | | -4.1 |
| *blr6718* |  | |  | hypothetical protein | 2.8 | | | | | | -6.9 |
| *blr6729* |  | |  | putative decarboxylase | 2.5 | | | | | | -3.3 |
| *blr6742* |  | |  | putative glutamate synthase small subunit | − | | | | | | − |
|  | *blr6743* | |  | putative ferredoxin oxidoreductase alpha subunit | 2.7 | | | | | | -2.4 |
|  | *blr6744* | |  | ferrodoxin oxidoreductase beta subunit | 3.1 | | | | | | -3.2 |
| *bll6746* |  | |  | hypothetical protein | − | | | | | | − |
|  | *bll6745* | |  | hypothetical protein | 3.9 | | | | | | -2.6 |
| *bll6756* |  | |  | hypothetical protein | − | | | | | | − |
|  | *bll6755* | |  | hypothetical protein | 5.2 | | | | | | -3.4 |
|  | *bll6754* | |  | hypothetical protein | 4.4 | | | | | | -5.7 |
| *blr6766* |  | |  | hypothetical protein | 2.5 | | | | | | -3.0 |
|  | *blr6767* | |  | trehalose synthase | 2.1 | | | | | | -4.0 |
|  | *blr6768* | | *glgB* | glycogen branching enzyme | − | | | | | | − |
|  | *blr6769* | | *glgX* | glycogen debranching enzyme | − | | | | | | − |
|  | *blr6770* | |  | alpha-amylase | − | | | | | | − |
|  | *blr6771* | |  | probable glycosyl hydrolase | − | | | | | | − |
| *bll6799* |  | |  | hypothetical protein | 10.0 | | | | | | -20.0 |
| *bll6995* |  | | *trmU* | tRNA (5-methylaminomethyl-2-thiouridylate)-methyltransferase | | | | | | − | − |
|  | *bll6994* | |  | putative phosphatidylethanolamine N-methyltransferase | | | | | | − | − |
|  | *bll6993* | |  | hypothetical protein | 8.9 | | | | | | -5.3 |
| *bll7010* |  | | *ssuD* | alkanesulfonate monooxygenase | − | | | | | | − |
|  | *bll7009* | |  | aliphatic sulfonate ABC transporter permease protein | 11.6 | | | | | | -6.5 |
|  | *bll7008* | |  | aliphatic sulfonate ABC transporter ATP-binding protein | | | | | 8.1 | | -3.5 |
|  | *bll7007* | |  | putative oxidoreductase | − | | | | | | − |
| *bll7022* |  | |  | hypothetical protein | − | | | | | | − |
|  | *bll7021* | |  | HlyD family secretion protein | − | | | | | | − |
|  | *bll7020* | |  | efflux protein | − | | | | | | − |
|  | *bll7019* | |  | AcrB/AcrD/AcrF family protein | − | | | | | | − |
|  | *bll7018* | |  | hypothetical protein | 9.5 | | | | | | -4.2 |
| *bsr7036* |  | | *napE* | periplasmic nitrate reductase protein | − | | | | | | − |
|  | *blr7037* | | *napD* | periplasmic nitrate reductase | − | | | | | | − |
|  | *blr7038* | | *napA* | periplasmic nitrate reductase large subunit precursor | − | | | | | | − |
|  | *blr7039* | | *napB* | periplasmic nitrate reductase small subunit precursor | 419.5 | | | | | | -2.8 |
|  | *blr7040* | | *napC* | cytochrome *c*-type protein | 130.4 | | | | | | -3.4 |
| *bsr7087* |  | |  | hypothetical protein | − | | | | | | − |
|  | *blr7088* | |  | hypothetical protein | 115.7 | | | | | | -3.4 |
| *blr7133* |  | |  | hypothetical protein | 9.7 | | | | | | -3.7 |
| *bll7252* |  | |  | hypothetical protein | 2.3 | | | | | | -2.2 |
| *bll7311* |  | |  | probable ArcD2 arginine/ornithine antiporter | 22.5 | | | | | | -12.5 |
|  | *bll7310* | |  | arginine deiminase | 15.6 | | | | | | -15.2 |
| *bll7313* |  | |  | RND efflux membrane fusion protein | 13.5 | | | | | | -5.0 |
|  | *bll7312* | |  | AcrB/AcrD/AcrF family protein | 15.3 | | | | | | -7.4 |
| *blr7314* |  | |  | hypothetical protein | 547.7 | | | | | | -93.5 |
|  | *blr7315* | |  | hypothetical protein | 23.4 | | | | | | -14.1 |
|  | *bsr7316* | |  | hypothetical protein | − | | | | | | − |
|  | *bsr7317* | |  | hypothetical protein | 4.6 | | | | | | -2.7 |
|  | *blr7318* | |  | hypothetical protein | 19.8 | | | | | | -2.1 |
|  | *blr7319* | |  | hypothetical protein | − | | | | | | − |
|  | *blr7320* | |  | hypothetical protein | 5.6 | | | | | | -3.5 |
| *blr7321* |  | |  | hypothetical protein | 321.1 | | | | | | -97.1 |
| *bll7322* |  | |  | hypothetical protein | 57.7 | | | | | | -17.6 |
| *blr7323* |  | |  | probable ArcD1 arginine/ornithine antiporter | 42.8 | | | | | | -8.3 |
| *blr7324* |  | |  | hypothetical protein | 4.4 | | | | | | -4.8 |
|  | *blr7325* | |  | hypothetical protein | 23.4 | | | | | | -9.4 |
|  | *blr7326* | |  | hypothetical protein | 22.9 | | | | | | -7.9 |
| *blr7327* |  | |  | hypothetical protein | 43.3 | | | | | | -17.2 |
| *bsr7328* |  | |  | hypothetical protein | 97.6 | | | | | | -46.7 |
| *blr7329* |  | |  | putative multidrug resistance protein | 5.5 | | | | | | -5.9 |
|  | *blr7330* | |  | AcrB/AcrD/AcrF family protein | 2.8 | | | | | | -2.9 |
| *bll7402* |  | | *serC* | phosphoserine aminotransferase | 2.3 | | | | | | -4.7 |
| *bll7411* |  | |  | hypothetical protein | 3.0 | | | | | | -6.4 |
| *bll7414* |  | |  | translation elongation factor EF-G | 8.1 | | | | | | -7.0 |
| *blr7436* |  | |  | hypothetical protein | 2.5 | | | | | | -5.9 |
| *bll7543* |  | | *glcD* | glycolate oxidase subunit | 2.7 | | | | | | -2.7 |
| *blr7544* |  | | *cycA* | cytochrome *c_550_* | 5.5 | | | | | | -3.3 |
| *bll7559* |  | | *chrC* | probable Fe/Mn superoxide dismutase | 8.6 | | | | | | -2.5 |
|  | *bll7558* | |  | hypothetical protein | − | | | | | | − |
| *blr7560* |  | | *dhlB* | 2-haloalkanoic acid dehalogenase | 9.2 | | | | | | -19.1 |
|  | *blr7561* | |  | hypothetical protein | − | | | | | | − |
| *bll7562* |  | |  | hypothetical protein | 7.8 | | | | | | -9.1 |
| *bsr7564* |  | |  | hypothetical protein | 97.6 | | | | | | -14.1 |
| *blr7621* |  | |  | hypothetical protein | 3.2 | | | | | | -2.0 |
| *blr7625* |  | |  | probable mandelate racemase | 3.4 | | | | | | -2.2 |
| *bll7626* |  | |  | hypothetical protein | 234.8 | | | | | | -27.4 |
| *bll7628* |  | |  | hypothetical protein | 49.6 | | | | | | -8.2 |
|  | *bll7627* | |  | hypothetical protein | 195.6 | | | | | | -10.7 |
| *blr7629* |  | |  | hypothetical protein | 140.7 | | | | | | -26.7 |
|  | *blr7630* | |  | probable decarboxylase | 26.6 | | | | | | -6.8 |
| *blr7631* |  | |  | putative MutT/nudix family protein | 5.3 | | | | | | -4.8 |
| *bsr7633* |  | |  | hypothetical protein bsr7633 | 9.1 | | | | | | -5.3 |
| *bll7635* |  | |  | hypothetical protein bll7635 | 3.2 | | | | | | -8.2 |
| *bll7638* |  | |  | putative cytochrome *c_6_* precursor | 3.5 | | | | | | -6.8 |
|  | *bll7637* | |  | hypothetical protein | − | | | | | | − |
|  | *bll7636* | |  | hypothetical protein | − | | | | | | − |
| *bll7648* |  | |  | hypothetical protein | 5.9 | | | | | | -4.6 |
| *bll7657* |  | |  | putative phosphoglycolate phosphatase | 3.2 | | | | | | -2.4 |
| *bll7672* |  | |  | putative protease secretion ATP-binding protein | − | | | | | | − |
|  | *bll7671* | |  | HlyD family secretion protein | − | | | | | | − |
|  | *bll7670* | |  | hypothetical protein | 4.6 | | | | | | -4.5 |
| *bll7763* |  | |  | acetoacetate decarboxylase | 2.5 | | | | | | -2.7 |
| *bll7774* |  | | *sodF* | superoxide dismutase | 2.6 | | | | | | -9.3 |
| *bll7790* |  | |  | hypothetical protein | 30.6 | | | | | | -10.3 |
| *bll7795* |  | | *phyR* | two-component response regulator | 4.1 | | | | | | -10.9 |
| *bsr7796* |  | | *nepR* | anti-sigma factor | 5.7 | | | | | | -6.5 |
|  | *blr7797* | | *ecfG* | RNA polymerase ECF sigma factor (σ^EcfG^) | 5.4 | | | | | | -3.7 |
| *blr7887* |  | |  | hypothetical protein | 4.3 | | | | | | -6.1 |
| *bll7908* |  | |  | hypothetical protein | − | | | | | | − |
|  | *bll7907* | |  | hypothetical protein | 2.1 | | | | | | -3.9 |
|  | *bll7906* | |  | putative ferredoxin | − | | | | | | − |
| *blr7909* |  | |  | hypothetical protein | 2.5 | | | | | | -2.1 |
| *bll7911* |  | |  | hypothetical protein | 5.0 | | | | | | -2.9 |
| *bll7952* |  | |  | probable selenium-binding protein | 4.2 | | | | | | -4.0 |
| *bll7960* |  | |  | hypothetical protein | 20.9 | | | | | | -3.9 |
| *bll7982* |  | |  | hypothetical protein | − | | | | | | − |
|  | *bll7981* | |  | putative dehydrogenase | 41.4 | | | | | | -4.2 |
| *bll8011* |  | |  | putative hydrolase | 2.7 | | | | | | -3.5 |
| *bll8024* |  | |  | hypothetical protein | − | | | | | | − |
|  | *bsl8023* | |  | hypothetical protein | 2.0 | | | | | | -2.4 |
| *bsr8030* |  | |  | hypothetical protein | 4.9 | | | | | | -2.3 |
| *bll8048* |  | |  | hypothetical protein | 17.9 | | | | | | -4.1 |
| *bll8143* |  | |  | hypothetical protein | 33.7 | | | | | | -4.0 |
|  | *bll8142* | |  | hypothetical protein | − | | | | | | − |
|  | |  |  |  |  | | | | | |  |
| **Class 2 (upregulated in the Δ*regR* strain)** | | | |  |  | | | | | |  |
| *bsl0098* |  | |  | hypothetical protein | 2.3 | | | | | | 4.3 |
| *bll0104* |  | | *gltP* | proton glutamate symport protein | 2.9 | | | | | | 2.9 |
|  | *bll0102* | | *gltI* | glutamate/aspartate periplasmic binding protein precursor | | | − | | | | − |
| *bsl0170* |  | |  | hypothetical protein | 6.0 | | | | | | 5.4 |
|  | *bsl0169* | |  | hypothetical protein | 17.3 | | | | | | 2.8 |
| *bsr0173* |  | |  | hypothetical protein | 5.0 | | | | | | 3.4 |
| *bll0182* |  | | *cisZ* | citrate synthase | 2.3 | | | | | | 2.0 |
| *blr0209* |  | | *comF* | competence protein F | 2.2 | | | | | | 2.4 |
| *bsr0210* |  | |  | glutaredoxin | 7.9 | | | | | | 2.2 |
|  | *blr0211* | |  | amidohydrolase | − | | | | | | − |
| *blr0230* |  | |  | probable esterase | 2.3 | | | | | | 2.2 |
| *bll0307* |  | |  | transcriptional regulatory protein | 2.4 | | | | | | 4.9 |
| *bll0330* |  | |  | two-component response regulator | 3.1 | | | | | | 2.9 |
| *blr0366* |  | |  | hypothetical protein | 18.1 | | | | | | 2.9 |
| *trnS-CGA* |  | |  | tRNA-Ser(CGA) | 3.7 | | | | | | 3.1 |
| *bsr0431* |  | |  | hypothetical protein | 10.2 | | | | | | 3.4 |
| *blr0488* |  | | *leuC* | isopropylmalate isomerase large subunit | 4.5 | | | | | | 5.0 |
| *bll0531* |  | |  | hypothetical protein | 11.6 | | | | | | 3.1 |
| *bsl0578* |  | |  | hypothetical protein | 9.8 | | | | | | 2.3 |
| *blr0586* |  | |  | hypothetical protein | 2.5 | | | | | | 2.6 |
| *bll0661* |  | |  | hypothetical protein | 2.4 | | | | | | 4.3 |
| *blr0675* |  | | *hrcA* | heat-inducible transcription repressor | 11.1 | | | | | | 3.1 |
| *bll0688* |  | |  | hypothetical protein | 2.0 | | | | | | 2.9 |
| *blr0694* |  | |  | probable peptidase | 2.0 | | | | | | 2.3 |
| *blr0709* |  | |  | hypothetical protein | 3.0 | | | | | | 2.3 |
| *bsl0728* |  | |  | hypothetical protein | 5.9 | | | | | | 2.1 |
| *bll0729* |  | | *hspH* | small heat shock protein | 7.4 | | | | | | 2.0 |
| *bll0777* |  | |  | transcriptional regulatory protein | 2.0 | | | | | | 6.1 |
|  | *bll0776* | |  | hypothetical protein | − | | | | | | − |
| *blr0850* |  | |  | hypothetical protein | 4.4 | | | | | | 3.5 |
|  | *blr0851* | |  | hypothetical protein | − | | | | | | − |
|  | *blr0852* | | *uppS* | undecaprenyl pyrophosphate synthase | − | | | | | | − |
| *blr0960* |  | |  | 5'-methylthioadenosine phosphorylase | 3.9 | | | | | | 3.5 |
|  | *blr0961* | |  | translation initiation factor IF-2B subunit alpha | 5.3 | | | | | | 5.7 |
| *bsl1006* |  | |  | hypothetical protein | 6.4 | | | | | | 2.3 |
| *blr1039* |  | |  | ABC transporter ATP-binding protein | − | | | | | | − |
|  | *bsr1040* | |  | hypothetical protein | 2.4 | | | | | | 4.6 |
|  | *blr1041* | |  | amidase | − | | | | | | − |
|  | *blr1042* | |  | hypothetical protein | − | | | | | | − |
|  | *blr1043* | |  | transcriptional regulatory protein | − | | | | | | − |
| *blr1072* |  | |  | hypothetical protein | 11.4 | | | | | | 8.1 |
| *blr1078* |  | |  | putative hydrolase | − | | | | | | − |
|  | *blr1079* | |  | hypothetical protein | 8.4 | | | | | | 2.7 |
| *trnG-CCC* |  | |  | tRNA-Gly(CCC) | 3.3 | | | | | | 3.0 |
| *bll1134* |  | |  | hypothetical protein | 3.7 | | | | | | 2.5 |
| *bll1135* |  | |  | transcriptional regulatory protein | 2.6 | | | | | | 3.1 |
| *bll1150* |  | |  | transcriptional regulatory protein | 14.6 | | | | | | 7.3 |
| *blr1221* |  | | *phnG* | phosphonate metabolism protein | 3.2 | | | | | | 3.8 |
|  | *blr1222* | | *phnH* | phosphonate metabolism protein | 2.8 | | | | | | 3.1 |
|  | *blr1223* | | *phnI* | phosphonate metabolism protein | 2.4 | | | | | | 3.5 |
|  | *blr1224* | | *phnJ* | phosphonate metabolism protein | − | | | | | | − |
|  | *blr1225* | | *phnK* | phosphonate uptake transporter ATP-binding protein | − | | | | | | − |
|  | *blr1226* | | *phnL* | phosphonate uptake transporter ATP-binding protein | − | | | | | | − |
|  | *blr1227* | | *phnM* | phosphonate metabolism protein | − | | | | | | − |
|  | *blr1228* | | *gmk* | guanylate kinase | − | | | | | | − |
|  | *blr1229* | |  | hypothetical protein | − | | | | | | − |
| *blr1288* |  | |  | probable long-chain-fatty-acid-CoA ligase | 12.2 | | | | | | 2.1 |
| *bll1295* |  | |  | probable oxidoreductase | 4.4 | | | | | | 2.6 |
|  | *bll1294* | |  | hypothetical protein | 2.3 | | | | | | 2.3 |
| *bll1367* |  | |  | hypothetical protein | 8.5 | | | | | | 2.5 |
|  | *bll1366* | |  | hypothetical protein | 2.1 | | | | | | 3.7 |
| *blr1375* |  | |  | hypothetical protein | − | | | | | | − |
|  | *blr1376* | |  | hypothetical protein blr1376 | 2.4 | | | | | | 2.3 |
| *blr1399* |  | | *metX* | homoserine O-acetyltransferase | 2.8 | | | | | | 2.0 |
|  | *blr1400* | |  | hypothetical protein | − | | | | | | − |
| *blr1404* |  | | *clpB* | ATP-dependent protease ATP-binding subunit | 6.8 | | | | | | 2.3 |
| *rrn16S* |  | |  | 16S rRNA | 8.6 | | | | | | 3.1 |
| *trnA-UGC* |  | |  | tRNA-Ala(TGC) | 2.9 | | | | | | 2.3 |
| *rrn23S* |  | |  | 23S rRNA | 8.2 | | | | | | 3.2 |
| *rrn5S* |  | |  | 5S rRNA | 8.4 | | | | | | 3.3 |
| *blr1477* |  | |  | probable trifunctional enzyme subunit | 16.4 | | | | | | 2.0 |
|  | *blr1478* | |  | hypothetical protein | − | | | | | | − |
|  | *blr1479* | |  | ferredoxin-nitrite reductase | − | | | | | | − |
|  | *blr1480* | |  | hypothetical protein | − | | | | | | − |
|  | *blr1481* | | *cysH* | phosphoadenosine phosphosulfate reductase | − | | | | | | − |
| *blr1506* |  | |  | hypothetical protein | 6.8 | | | | | | 3.0 |
| *bll1688* |  | |  | probable suppressor protein | 4.7 | | | | | | 2.4 |
|  | *bll1687* | |  | hypothetical protein | − | | | | | | − |
| *blr2071* |  | |  | similar to inosamine-phosphate amidinotransferas | 9.1 | | | | | | 5.5 |
| *bsr2110* |  | |  | hypothetical protein | 8.5 | | | | | | 5.1 |
|  | *bsr2111* | |  | hypothetical protein | − | | | | | | − |
| *blr2113* |  | |  | hypothetical protein | − | | | | | | − |
|  | *blr2114* | |  | hypothetical protein | 2.5 | | | | | | 5.5 |
|  | *blr2115* | |  | hypothetical protein | − | | | | | | − |
| *bll2215* |  | |  | hypothetical protein | 4.5 | | | | | | 2.4 |
| *bll2284* |  | |  | hypothetical protein | 3.9 | | | | | | 3.3 |
| *blr2286* |  | |  | hypothetical protein | 7.3 | | | | | | 4.8 |
|  | *blr2287* | |  | two-component hybrid sensor and regulator | − | | | | | | − |
|  | *blr2288* | |  | two-component hybrid sensor and regulator | − | | | | | | − |
| *blr2325* |  | |  | transcriptional regulatory protein | 2.3 | | | | | | 2.9 |
| *bll2327* |  | | *pncB* | nicotinate phosphoribosyltransferase | 2.6 | | | | | | 2.1 |
| *bll2330* |  | |  | hypothetical protein | 10.5 | | | | | | 3.6 |
|  | *bll2329* | |  | hypothetical protein | 3.0 | | | | | | 2.1 |
|  | *bsl2328* | |  | hypothetical protein | − | | | | | | − |
| *bll2336* |  | |  | transcriptional regulatory protein | 7.2 | | | | | | 5.0 |
| *bll2417* |  | |  | hypothetical protein | 4.3 | | | | | | 2.7 |
| *bsl2435* |  | |  | hypothetical protein | 2.5 | | | | | | 3.3 |
|  | *bll2434* | |  | plasmid stability protein | − | | | | | | − |
| *blr2456* |  | |  | hypothetical protein | 6.6 | | | | | | 2.2 |
| *bll2516* |  | |  | hypothetical protein | − | | | | | | − |
|  | *bll2515* | |  | similar to pyruvate phosphate dikinase | − | | | | | | − |
|  | *bll2514* | |  | hypothetical protein | − | | | | | | − |
|  | *bll2513* | |  | hypothetical protein | 2.3 | | | | | | 2.2 |
| *bsr2531* |  | |  | hypothetical protein | 4.3 | | | | | | 2.4 |
|  | *blr2533* | |  | hypothetical protein | − | | | | | | − |
| *bll2532* |  | | *ispB* | octaprenyl-diphosphate synthase | 3.1 | | | | | | 2.0 |
| *blr2579* |  | |  | hypothetical protein | 6.2 | | | | | | 4.1 |
| *bll2590* |  | |  | hypothetical protein | 115.2 | | | | | | 3.2 |
| *bll2604* |  | |  | transcriptional regulatory protein | 2.5 | | | | | | 4.8 |
| *blr2607* |  | |  | hypothetical protein | 10.1 | | | | | | 2.5 |
| *bll2628* |  | | *prtI* | ECF family sigma factor | 2.7 | | | | | | 3.8 |
| *trnQ-UUG* |  | |  | tRNA-Gln(TTG) | 2.7 | | | | | | 3.1 |
| *bsr2670* |  | |  | hypothetical protein | 79.4 | | | | | | 2.2 |
| *bsr2672* |  | |  | hypothetical protein | 33.5 | | | | | | 4.3 |
| *bll2683* |  | |  | hypothetical protein | − | | | | | | − |
|  | *bll2682* | | *macA* | maleylacetate reductase | 4.3 | | | | | | 3.0 |
|  | *bll2681* | |  | hypothetical protein | − | | | | | | − |
|  | *bll2680* | |  | probable dehydrogenase | − | | | | | | − |
|  | *bll2679* | |  | dioxygenase | − | | | | | | − |
| *blr2702* |  | |  | hypothetical protein | 5.3 | | | | | | 2.2 |
| *bll2757* |  | | *fixK_2_* | transcriptional regulator FixK_2_ | 17.9 | | | | | | 2.2 |
| *bll2758* |  | |  | two-component response regulator | 38.8 | | | | | | 2.6 |
| *blr2761* |  | |  | hypothetical protein | 77.3 | | | | | | 3.4 |
| *blr2762* |  | |  | hypothetical protein | 39.4 | | | | | | 3.1 |
| *bll2855* |  | | *rocD* | ornithine--oxo-acid transaminase | 10.5 | | | | | | 3.7 |
| *bsr2878* |  | |  | hypothetical protein | 2.6 | | | | | | 4.2 |
|  | *blr2879* | |  | hypothetical protein | − | | | | | | − |
| *blr2887* |  | |  | hypothetical protein | 31.0 | | | | | | 3.1 |
| *blr2912* |  | |  | probable ABC transporter permease protein | 2.9 | | | | | | 2.1 |
|  | *blr2913* | |  | probable ABC transporter permease protein | − | | | | | | − |
| *blr2921* |  | |  | hypothetical protein | 4.8 | | | | | | 5.6 |
|  | *blr2922* | |  | ABC transporter amino acid-binding protein | − | | | | | | − |
| *blr2987* |  | |  | hypothetical protein | 45.3 | | | | | | 2.2 |
| *blr2988* |  | |  | hypothetical protein | 4.0 | | | | | | 2.4 |
| *bsl3053* |  | |  | hypothetical protein | 2.2 | | | | | | 2.7 |
| *blr3067* |  | |  | hypothetical protein | 2.5 | | | | | | 2.3 |
| *bll3090* |  | |  | transcriptional regulatory protein | 3.1 | | | | | | 3.7 |
|  | *bll3089* | |  | hypothetical protein | − | | | | | | − |
|  | *bll3088* | |  | hypothetical protein | − | | | | | | − |
| *blr3091* |  | |  | transcriptional regulatory protein | 8.3 | | | | | | 2.4 |
| *blr3092* |  | |  | putative secreted protein | 2.2 | | | | | | 3.1 |
|  | *blr3093* | |  | hypothetical protein | − | | | | | | − |
| *bll3117* |  | |  | putative thymidine phosphorylase | 7.3 | | | | | | 3.1 |
|  | *bll3116* | |  | phosphoribosylpyrophosphate synthetase | − | | | | | | − |
|  | *bll3115* | |  | hypothetical protein | − | | | | | | − |
| *bll3177* |  | |  | probable arginine/lysine/ornithine decarboxylase | − | | | | | | − |
|  | *bll3176* | |  | acetyltransferase | 7.7 | | | | | | 2.4 |
|  | *bsl3175* | |  | hypothetical protein | 35.8 | | | | | | 2.1 |
| *bll3434* |  | |  | transcriptional regulatory protein MarR family | 3.0 | | | | | | 2.2 |
| *blr3455* |  | |  | hypothetical protein | 3.0 | | | | | | 2.2 |
| *bll3471* |  | |  | hypothetical membrane protein | − | | | | | | − |
|  | *bll3470* | |  | hypothetical protein | − | | | | | | − |
|  | *bll3469* | |  | hypothetical protein | 5.0 | | | | | | 2.2 |
| *blr3479* |  | |  | hypothetical protein | 9.6 | | | | | | 4.4 |
| *bll3507* |  | |  | hypothetical protein | 3.3 | | | | | | 2.5 |
| *blr3521* |  | |  | hypothetical protein | 12.8 | | | | | | 4.9 |
| *blr3585* |  | |  | hypothetical protein | 2.5 | | | | | | 2.0 |
| *blr3741* |  | |  | hypothetical protein | 2.1 | | | | | | 5.8 |
|  | *blr3742* | | *mrp* | probable multidrug-resistance related protein | − | | | | | | − |
| *bsl3746* |  | |  | hypothetical protein | 2.1 | | | | | | 3.4 |
| *bll3785* |  | | *coxM* | cytochrome *c* oxidase | 27.2 | | | | | | 5.7 |
|  | *bll3784* | | *coxN* | cytochrome *c* oxidase | 15.4 | | | | | | 3.7 |
|  | *bll3783* | | *coxO* | cytochrome *c* oxidase | 8.9 | | | | | | 3.0 |
|  | *bll3782* | | *coxP* | cytochrome *c* oxidase | − | | | | | | − |
|  | *bll3781* | |  | hypothetical protein | − | | | | | | − |
| *blr3787* |  | |  | hypothetical protein | 22.3 | | | | | | 5.0 |
| *blr3799* |  | |  | probable oxidoreductase | 3.6 | | | | | | 2.4 |
| *bll3835* |  | |  | hypothetical protein | 40.5 | | | | | | 2.2 |
| *blr3873* |  | |  | transcriptional regulatory protein | 2.6 | | | | | | 2.5 |
| *bll3877* |  | |  | transcriptional regulatory protein | 3.2 | | | | | | 3.8 |
|  | *bll3876* | |  | aldehyde dehydrogenase | − | | | | | | − |
|  | *bll3875* | |  | hypothetical metabolite transport protein | − | | | | | | − |
| *trnQ-CUG* |  | |  | tRNA-Gln(CTG) | 6.5 | | | | | | 2.8 |
| *blr3963* |  | |  | transcriptional regulatory protein | 2.7 | | | | | | 9.0 |
| *bll3994* |  | |  | hypothetical protein | − | | | | | | − |
|  | *bll3993* | |  | hypothetical protein | 18.5 | | | | | | 2.4 |
| *blr3995* |  | |  | hypothetical protein | 10.8 | | | | | | 3.5 |
| *bsr3996* |  | |  | hypothetical protein | 9.0 | | | | | | 4.7 |
| *blr3997* |  | |  | hypothetical protein | 2.1 | | | | | | 2.6 |
| *blr4111* |  | |  | hypothetical protein | 25.9 | | | | | | 3.4 |
|  | *blr4112* | |  | probale cation efflux system protein | 2.5 | | | | | | 2.3 |
|  | *blr4113* | |  | hypothetical protein | − | | | | | | − |
| *blr4114* |  | |  | hypothetical protein | 110.3 | | | | | | 2.2 |
|  | *blr4115* | | *actP* | acetate permease | − | | | | | | − |
| *blr4131* |  | |  | hypothetical protein | 6.7 | | | | | | 3.6 |
|  | *blr4132* | |  | hypothetical protein | 9.2 | | | | | | 2.2 |
| *blr4162* |  | |  | hypothetical protein | 47.8 | | | | | | 2.6 |
| *bll4168* |  | |  | hypothetical protein | − | | | | | | − |
| *bsr4175* |  | |  | hypothetical protein | 33.5 | | | | | | 2.2 |
| *bll4189* |  | |  | putative acetyl-hydrolase | 2.6 | | | | | | 2.9 |
| *blr4224* |  | |  | hypothetical protein | 54.4 | | | | | | 2.7 |
| *bsr4236* |  | |  | hypothetical protein | 63.7 | | | | | | 2.3 |
| *blr4240* |  | |  | hypothetical protein | 97.9 | | | | | | 3.5 |
|  | *blr4241* | |  | hypothetical protein | 254.2 | | | | | | 2.6 |
| *blr4242* |  | |  | hypothetical protein | 12.7 | | | | | | 3.0 |
| *bsr4244* |  | |  | hypothetical protein | 4.2 | | | | | | 4.2 |
| *blr4300* |  | |  | probable DNA-binding protein | 3.0 | | | | | | 2.4 |
| *bll4303* |  | |  | putative amidase | 2.0 | | | | | | 2.5 |
| *bll4574* |  | |  | hypothetical protein | 3.3 | | | | | | 2.3 |
| *blr4630* |  | |  | hypothetical protein | 4.1 | | | | | | 2.0 |
| *blr4637* |  | |  | probable HspC2 heat shock protein | 143.1 | | | | | | 2.3 |
| *blr4646* |  | |  | hypothetical protein | 160.3 | | | | | | 2.5 |
| *bll4651* |  | |  | hypothetical protein | 180.5 | | | | | | 2.3 |
| *blr4652* |  | |  | hypothetical protein | 248.7 | | | | | | 2.2 |
|  | *blr4653* | | *dnaJ* | molecular chaperone DnaJ family | − | | | | | | − |
|  | *blr4654* | |  | hypothetical protein | − | | | | | | − |
| *bsr4666* |  | |  | hypothetical protein | 2.1 | | | | | | 3.1 |
| *bsl4703* |  | |  | hypothetical protein | 4.8 | | | | | | 4.2 |
| *bll4718* |  | |  | hypothetical protein | 56.0 | | | | | | 3.5 |
| *bsr4726* |  | |  | hypothetical protein | 35.5 | | | | | | 2.9 |
| *bll4785* |  | |  | transcriptional regulatory protein | 2.6 | | | | | | 3.1 |
| *blr4890* |  | |  | hypothetical protein | 4.2 | | | | | | 2.7 |
| *trnD-GUC* |  | |  | tRNA-Asp(GTC) | 5.2 | | | | | | 2.8 |
| *bll4952* |  | |  | NfeD protein homolog | 14.0 | | | | | | 4.0 |
|  | *bll4951* | |  | putative stomatin-like protein | 22.2 | | | | | | 4.0 |
| *blr4955* |  | |  | putative cytochrome *b_561_* | 48.0 | | | | | | 3.1 |
| *bsr4956* |  | |  | hypothetical protein | 2.8 | | | | | | 5.6 |
| *bll4998* |  | |  | hypothetical protein | 2.4 | | | | | | 3.3 |
|  | *bll4997* | |  | hypothetical protein bll4997 | − | | | | | | − |
| *trnP-GGG* |  | |  | tRNA-Pro(GGG) | 3.5 | | | | | | 2.8 |
| *bll5019* |  | | *ihfA* | integration host factor alpha subunit | 4.0 | | | | | | 2.1 |
|  | *bll5018* | |  | hypothetical protein | − | | | | | | − |
| *blr5037* |  | | *hemB* | delta-aminolevulinic acid dehydratase | 4.9 | | | | | | 2.4 |
| *bll5044* |  | | *mntH* | putative manganese transport protein MntH | 2.2 | | | | | | 2.2 |
| *blr5051* |  | |  | superoxide dismutase SodM-like protein | 3.7 | | | | | | 2.9 |
|  | *blr5052* | |  | putative chromate transport protein | − | | | | | | − |
| *trnS-GCU* |  | |  | tRNA-Ser(GCT) | 5.7 | | | | | | 3.2 |
| *bsl5090* |  | | *gatC* | glutamyl-tRNA-Gln-amidotransferase chain C | 3.7 | | | | | | 2.9 |
| *blr5118* |  | |  | hypothetical protein | 7.0 | | | | | | 3.3 |
| *bll5146* |  | |  | hypothetical protein | 3.1 | | | | | | 3.6 |
|  | *bll5145* | |  | probable mannose-6-phosphate isomerase | − | | | | | | − |
| *blr5150* |  | |  | hypothetical protein | 30.4 | | | | | | 3.0 |
| *blr5151* |  | |  | hypothetical transport protein | 3.1 | | | | | | 3.5 |
| *bll5155* |  | |  | hypothetical protein | 8.9 | | | | | | 2.5 |
| *bll5160* |  | |  | hypothetical protein | 6.3 | | | | | | 2.9 |
| *bll5164* |  | |  | hypothetical protein | 2.1 | | | | | | 5.8 |
| *bsl5165* |  | |  | hypothetical protein | 6.9 | | | | | | 2.6 |
| *bll5199* |  | |  | hypothetical protein | 4.2 | | | | | | 5.1 |
| *bll5219* |  | | *hspD* | small heat shock protein | 2.6 | | | | | | 2.1 |
| *blr5233* |  | | *hspB* | small heat shock protein | 9.6 | | | | | | 2.2 |
|  | *blr5234* | | *hspC* | small heat shock protein | 2.3 | | | | | | 2.3 |
| *bll5249* |  | |  | oxidoreductase | 3.5 | | | | | | 4.3 |
| *bsr5273* |  | |  | hypothetical protein | 810.9 | | | | | | 3.5 |
| *bll5296* |  | |  | hypothetical protein | 4.7 | | | | | | 2.6 |
| *blr5308* |  | |  | anti-oxidant protein | 8.5 | | | | | | 2.2 |
| *bll5354* |  | |  | probable transmembrane protein | 5.7 | | | | | | 3.2 |
| *bsl5473* |  | |  | hypothetical protein | 10.8 | | | | | | 4.3 |
| *blr5525* |  | |  | hypothetical protein | 5.3 | | | | | | 2.1 |
| *bll5551* |  | |  | hypothetical protein | 4.9 | | | | | | 2.7 |
| *bll5589* |  | |  | hypothetical protein | 3.6 | | | | | | 2.4 |
|  | *bll5588* | |  | hypothetical protein | − | | | | | | − |
| *trnN-GUU-2* |  | |  | tRNA-Asn(GTT) | 3.3 | | | | | | 6.4 |
| *trnF-GAA-2* |  | |  | tRNA-Phe(GAA) | 3.6 | | | | | | 5.4 |
| *bll5651* |  | |  | transcriptional regulatory protein | 5.7 | | | | | | 2.3 |
|  | *bll5650* | |  | hypothetical protein | − | | | | | | − |
|  | *bll5649* | |  | ABC transporter ATP-binding protein | − | | | | | | − |
|  | *bll5648* | |  | nitrate ABC transporter permease protein | − | | | | | | − |
| *bll5662* |  | |  | hypothetical protein | − | | | | | | − |
|  | *bll5661* | |  | hypothetical protein | 3.8 | | | | | | 2.1 |
|  | *bll5660* | |  | hypothetical protein | − | | | | | | − |
|  | *bll5659* | |  | hypothetical protein | − | | | | | | − |
| *bll5665* |  | | *cooxS* | putative carbon monoxide dehydrogenase small subunit | | 2.9 | | | | | 4.1 |
|  | *bll5664* | | *cooxM* | putative carbon monoxide dehydrogenase medium subunit | | | − | | | | − |
| *blr5687* |  | |  | two-component sensor histidine kinase | 4.7 | | | | | | 2.7 |
| *bll5711* |  | | *grlA* | glutaredoxin-related protein | 5.0 | | | | | | 2.0 |
|  | *bll5710* | |  | hypothetical protein | − | | | | | | − |
|  | *bll5709* | |  | hypothetical protein | − | | | | | | − |
|  | *bll5708* | |  | hypothetical protein | − | | | | | | − |
| *bll5729* |  | | *purC* | phosphoribosylaminoimidazole-succinocarboxamide synthase | | | | − | | | − |
|  | *bsl5728* | |  | phosphoribosylformylglycinamidine synthase | 3.7 | | | | | | 2.1 |
| *blr5735* |  | |  | transcriptional regulatory protein | 4.0 | | | | | | 6.6 |
| *bll5764* |  | |  | hypothetical protein | 7.5 | | | | | | 3.9 |
| *bll5772* |  | |  | hypothetical protein | 32.1 | | | | | | 3.2 |
|  | *bll5771* | |  | AcrB/AcrD/AcrF family cation efflux protein | − | | | | | | − |
|  | *bll5770* | |  | hypothetical protein | − | | | | | | − |
| *bll5773* |  | |  | transcriptional regulatory protein | 7.9 | | | | | | 2.0 |
| *blr5774* |  | |  | probable sulfide-quinone reductase | 12.3 | | | | | | 3.7 |
|  | *blr5775* | |  | putative thioredoxin | 10.0 | | | | | | 2.8 |
|  | *bsr5776* | |  | hypothetical protein | 28.0 | | | | | | 2.6 |
| *blr5777* |  | |  | hypothetical protein | 10.7 | | | | | | 2.4 |
|  | *blr5778* | | *fixG* | nitrogen fixation protein | − | | | | | | − |
| *bll5780* |  | |  | similar to FrnE protein | 2.1 | | | | | | 2.7 |
| *bsr5798* |  | |  | hypothetical protein | 15.1 | | | | | | 2.4 |
| *blr5858* |  | |  | hypothetical protein | 3.1 | | | | | | 2.4 |
| *blr5860* |  | |  | transcriptional regulatory protein | 6.2 | | | | | | 9.9 |
| *bll5941* |  | |  | putative partition protein | 9.2 | | | | | | 4.1 |
|  | *bll5940* | |  | hypothetical protein | 7.6 | | | | | | 3.2 |
| *bll5959* |  | |  | hypothetical protein | − | | | | | | − |
|  | *bll5958* | |  | hypothetical protein | − | | | | | | − |
|  | *bll5957* | |  | hypothetical protein | 7.0 | | | | | | 2.3 |
| *bll6051* |  | |  | hypothetical protein | 3.1 | | | | | | 3.5 |
|  | *bll6050* | |  | hypothetical protein | − | | | | | | − |
|  | *bsl6049* | |  | hypothetical protein | − | | | | | | − |
| *bll6061* |  | | *fixK_1_* | transcriptional regulatory protein | 61.8 | | | | | | 3.0 |
| *bll6069* |  | |  | hypothetical protein | 97.4 | | | | | | 2.3 |
| *bll6076* |  | |  | putative acetyl-CoA synthetase | 2.4 | | | | | | 2.1 |
|  | *bll6075* | |  | hypothetical protein | − | | | | | | − |
| *bll6077* |  | |  | transcriptional regulatory protein | 2.1 | | | | | | 2.6 |
| *blr6078* |  | |  | probable substrate-binding protein | 2.4 | | | | | | 2.5 |
|  | *blr6079* | |  | hypothetical protein | − | | | | | | − |
| *bll6110* |  | |  | hypothetical protein | 2.7 | | | | | | 8.2 |
| *bsr6229* |  | |  | hypothetical protein | 3.0 | | | | | | 2.0 |
| *blr6259* |  | |  | transcriptional regulatory protein | 4.2 | | | | | | 4.2 |
|  | *bll6260* | |  | methionine sulfoxide reductase A | − | | | | | | − |
| *bll6264* |  | |  | putative hydrolase | − | | | | | | − |
|  | *bll6263* | |  | putative enoyl-CoA hydratase | 2.9 | | | | | | 2.7 |
| *blr6277* |  | |  | transcriptional regulatory protein | 3.6 | | | | | | 2.9 |
| *blr6291* |  | |  | transcriptional regulatory protein | 3.3 | | | | | | 2.2 |
| *blr6408* |  | |  | transcriptional regulatory protein | 2.7 | | | | | | 3.1 |
| *ssrA2* |  | |  | tmRNA-coding_RNA | 7.0 | | | | | | 3.3 |
| *bll6552* |  | |  | hypothetical protein | 12.4 | | | | | | 2.2 |
| *blr6553* |  | |  | transcriptional regulatory protein | 3.3 | | | | | | 4.8 |
| *bll6613* |  | |  | hypothetical protein | 3.5 | | | | | | 2.2 |
|  | *bll6612* | |  | hypothetical protein | − | | | | | | − |
| *rnpB* |  | |  | RNase P subunit B | 7.6 | | | | | | 3.0 |
| *bll6662* |  | | *rdh* | ribitol 2-dehydrogenase | 2.4 | | | | | | 2.6 |
| *bll6670* |  | |  | hypothetical protein | 3.1 | | | | | | 2.0 |
| *bll6673* |  | |  | hypothetical protein | 17.7 | | | | | | 2.6 |
| *blr6829* |  | |  | transcriptional regulatory protein | 2.3 | | | | | | 2.4 |
| *bll6893* |  | |  | hypothetical protein | 29.8 | | | | | | 3.9 |
| *blr6907* |  | |  | hypothetical protein | 3.0 | | | | | | 3.1 |
| *bll6937* |  | | *hupG* | HupG protein | 12.7 | | | | | | 2.3 |
|  | *bll6936* | | *hupH* | HupH protein | − | | | | | | − |
|  | *bsl6935* | | *hupI* | HupI protein | − | | | | | | − |
|  | *bll6934* | | *hupJ* | HupJ protein | − | | | | | | − |
|  | *bll6933* | | *hupK* | HupK protein | − | | | | | | − |
|  | *bll6932* | | *hypA* | HypA protein | − | | | | | | − |
|  | *bll6931* | | *hypB* | HypB protein | − | | | | | | − |
|  | *bll6930* | | *hypF* | hydrogenase maturation protein | − | | | | | | − |
|  | *bsl6929* | | *hypC* | hydrogenase expression/formation protein | − | | | | | | − |
|  | *bll6928* | | *hypD'* | HypD' protein | − | | | | | | − |
|  | *bll6927* | | *hypE* | HypE protein | − | | | | | | − |
|  | *bll6926* | | *hoxX* | probable sensor protein | − | | | | | | − |
|  | *bll6925* | |  | two-component response regulator | − | | | | | | − |
|  | *bll6924* | |  | two-component hybrid sensor and regulator | − | | | | | | − |
| *bll7034* |  | |  | MDO-like protein | 2.4 | | | | | | 2.7 |
| *bll7035* |  | |  | transcriptional regulatory protein | 3.8 | | | | | | 3.4 |
| *bll7046* |  | |  | hypothetical protein | 3.6 | | | | | | 2.5 |
| *blr7054* |  | |  | hypothetical protein | 16.0 | | | | | | 3.3 |
| *bll7059* |  | |  | hypothetical protein | 2.8 | | | | | | 3.5 |
| *blr7084* |  | | *nnrR* | transcriptional regulatory protein | 13.7 | | | | | | 2.9 |
| *bsr7110* |  | |  | hypothetical protein | 3.9 | | | | | | 2.9 |
| *bll7113* |  | |  | hypothetical protein | 2.6 | | | | | | 2.3 |
| *bll7160* |  | |  | hypothetical protein | 8.0 | | | | | | 2.6 |
| *bll7164* |  | |  | hypothetical protein | 9.0 | | | | | | 2.9 |
| *blr7208* |  | |  | transcriptional regulatory protein | 3.6 | | | | | | 3.5 |
| *bsr7215* |  | |  | hypothetical protein | 4.2 | | | | | | 3.7 |
| *bll7217* |  | |  | probable site-specific integrase/recombinase | 4.0 | | | | | | 2.3 |
| *bll7221* |  | |  | hypothetical protein | 7.7 | | | | | | 4.3 |
| *blr7228* |  | |  | hypothetical protein | 23.7 | | | | | | 2.9 |
| *blr7261* |  | | *putA* | proline dehydrogenase | 2.5 | | | | | | 3.9 |
|  | *blr7262* | |  | putative racemase | − | | | | | | − |
| *blr7345* |  | |  | hypothetical protein | 72.0 | | | | | | 3.1 |
| *bll7347* |  | |  | hypothetical protein | 8.3 | | | | | | 2.2 |
| *blr7381* |  | | *trxB* | thioredoxin reductase | 3.6 | | | | | | 2.6 |
| *bsr7426* |  | |  | hypothetical protein | 3.0 | | | | | | 2.6 |
| *bsl7442* |  | |  | hypothetical protein | 3.2 | | | | | | 3.5 |
| *bsr7468* |  | | *cspA* | cold shock protein | 3.7 | | | | | | 2.2 |
| *bll7513* |  | |  | hypothetical protein | 2.8 | | | | | | 2.0 |
| *blr7552* |  | |  | hypothetical protein | 6.5 | | | | | | 2.1 |
| *bll7565* |  | |  | transcriptional regulatory protein | 3.3 | | | | | | 2.1 |
| *bsr7705* |  | |  | hypothetical protein | 3.2 | | | | | | 2.1 |
|  | *blr7706* | |  | hypothetical protein | − | | | | | | − |
| *bsr7707* |  | |  | hypothetical protein | 3.9 | | | | | | 2.2 |
| *blr7716* |  | |  | probable adenylate cyclase | 4.3 | | | | | | 2.1 |
|  | *blr7717* | |  | putative adenylate cyclase | − | | | | | | − |
| *blr7740* |  | |  | small heat shock protein | 22.9 | | | | | | 3.4 |
| *bll7749* |  | |  | hypothetical protein | 2.8 | | | | | | 3.7 |
| *bll7750* |  | |  | hypothetical protein | 3.1 | | | | | | 3.9 |
| *bsl7781* |  | |  | hypothetical protein | 34.2 | | | | | | 3.1 |
| *bll7787* |  | |  | hypothetical protein | 64.0 | | | | | | 2.9 |
| *blr7813* |  | |  | transcriptional regulatory protein | 2.7 | | | | | | 2.1 |
|  | *blr7814* | |  | putative L-proline 4-hydroxylase | − | | | | | | − |
| *bll7838* |  | | *pobA* | 4-hydroxybenzoate 3-monooxygenase | 3.0 | | | | | | 2.1 |
| *blr7881* |  | |  | transcriptional regulatory protein | 9.0 | | | | | | 4.1 |
| *bll7941* |  | |  | aminopeptidase | 4.6 | | | | | | 2.4 |
| *blr7984* |  | |  | transcriptional regulatory protein | 8.4 | | | | | | 4.1 |
| *bsl7992* |  | |  | hypothetical protein | 119.5 | | | | | | 3.4 |
|  | *bll7991* | |  | hypothetical protein | 88.6 | | | | | | 2.8 |
|  | *bll7990* | |  | hypothetical protein | − | | | | | | − |
| *bll8020* |  | |  | putative ubiquinone/menaquinone biosynthesis methyltransferase | | | | | 2.7 | | 3.4 |
| *blr8039* |  | |  | hypothetical protein | 4.2 | | | | | | 2.0 |

^a^  *B. japonicum* wild-type and Δ*regR* (strain 2426) cells were grown under anoxic conditions (with nitrate as terminal electron acceptor) in Bergersen minimal medium with succinate as carbon source (BSN). Anoxically induced genes in the wild type were identified using as reference wild-type cells grown oxically in PSY medium [33].

^b^ Genes numbers are according to the Rhizobase (http://genome.kazusa.or.jp/rhizobase/).

^c^ Operon predictions are those described by Hauser *et al*. (2007) [22] and Mesa *et al*. (2008) [14]. All putative operon members, although not controlled in the selected conditions, are included in the Table.

^d^ Genes names as indicated in the EMBL-EBI database with modifications.

^e^ Protein description according to Kaneko *et al*. (2002) [67] with modifications.

^f^ Gene expression changes (*n* fold) determined by microarray analyses. Negative values indicate decrease of expression; (−) indicates the gene was not differentially expressed.

^g^ Fold change of expression of upregulated genes in a comparison of wild-type cells grown anoxically in BSN medium with those grown oxically in PSY medium.

^h^ Fold change of expression by comparison of Δ*regR* cells with wild type cells. both grown anoxically.

^i^ blr2806, blr2807, blr2808 and blr2809 have been shown to form an operon (J. Cabrera and M.J. Delgado, unpublished results), and blr2807 has been recently identified as a haemoglobin and named as *bjgb* [44].
