# Supplementary material for: The Global Response Regulator RegR Controls Expression of Denitrification Genes in Bradyrhizobium japonicum
Source: PLoS One. 2014 Jun 20;9(6):e99011. doi: 10.1371/journal.pone.0099011 (PMC4064962; doi:10.1371/journal.pone.0099011)
Supplement: Table S3 — Differentially expressed genes by a factor of ≤−5 or ≥5 in the Δ regR strain grown anoxically and their putative operon members. (DOCX) [file pone.0099011.s003.docx]

Table S3: Differentially expressed genes by a factor of ≤ -5 or ≥ 5 in the Δ*regR* strain grown anoxically and their putative operon members^a^

| **Class and gene no.^b^** | **Putative operon member (gene no.^)c^** | **Gene name^d^** | **Description^e^** | **Fold change^f^** | |
| --- | --- | --- | --- | --- | --- |
| **Class 1 (downregulated in the Δr*egR* strain)** | |  |  | |  |
| *bll0091* |  |  | ABC transporter substrate-binding protein | | − |
|  | *bll0090* |  | ABC transporter ATP-binding protein | | − |
|  | *bll0089* |  | ABC transporter permease protein | | − |
|  | *bll0088* |  | glycerate dehydrogenase | | -5.1 |
|  | *bll0087* |  | hypothetical protein | | − |
| *blr0149* |  | *cyoA* | cytochrome *o* ubiquinol oxidase subunit II | | -6.0 |
|  | *blr0150* | *cyoB* | cytochrome *o* ubiquinol oxidase subunit I | | -4.4 |
|  | *blr0151* | *cyoC* | cytochrome *o* ubiquinol oxidase subunit III | | -6.8 |
|  | *blr0152* | *cyoD* | cytochrome *o* ubiquinol oxidase subunit IV | | -4.7 |
|  | *blr0153* |  | probable surfeit locus protein 1 | | -4.6 |
|  | *blr0154* |  | two-component sensor histidine kinase | | − |
|  | *blr0155* |  | two-component response regulator | | − |
| *bll0233* |  |  | hypothetical protein | | -18.1 |
| *bll0246* |  | *bam* | amidase | | -6.1 |
| *blr0274* |  |  | hypothetical protein | | -12.7 |
| *blr0305* |  |  | hypothetical protein | | -9.5 |
| *blr0314* |  | *nosR* | nitrous oxide reductase expression regulator | | -2.2 |
|  | *blr0315* | *nosZ* | nitrous-oxide reductase precursor | | -7.6 |
|  | *blr0316* | *nosD* | periplasmic copper-binding precursor | | -12.1 |
|  | *blr0317* | *nosF* | copper ABC transporter | | -14.2 |
|  | *blr0318* | *nosY* | nitrous oxide metabolic protein | | -10.5 |
|  | *blr0319* | *nosL* | NosL protein | | -11.1 |
|  | *blr0320* | *nosX* | NosX protein | | -10.5 |
| *bll0322* |  | *otsA* | probable trehalose-6-phosphate synthase | | -5.5 |
| *bll0342* |  | *fah* | fumarylacetoacetase | | -8.6 |
| *bll0346* |  |  | putative oxidoreductase | | − |
|  | *bsl0345* |  | hypothetical protein | | − |
|  | *bll0344* |  | hypothetical protein | | − |
|  | *bll0343* |  | homogentisate 1.2-dioxygenase | | -9.6 |
| *blr0401* |  |  | hypothetical protein | | -5.6 |
| *blr0420* |  | *rplU* | 50S ribosomal protein L21 | | -5.8 |
| *blr0444* |  |  | - | | -7.5 |
| *bll0465* |  |  | hypothetical protein | | -10.6 |
| *blr0495* |  | *leuD* | isopropylmalate isomerase small subunit | | -5.6 |
| *bll0506* |  |  | hypothetical protein | | -4.8 |
|  | *bll0505* |  | hypothetical protein | | -5.4 |
| *blr0583* |  | *ggt* | gamma-glutamyltranspeptidase | | -5.9 |
| *bll0598* |  |  | hypothetical protein | | -11.0 |
| *bll0633* |  | *gidA* | glucose-inhibited division protein A | | -6.7 |
|  | *bll0632* | *gidB* | probable methyltransferase | | − |
|  | *bll0631* | *parA* | chromosome partitioning protein A | | 3.5 |
| *blr0651* |  |  | hypothetical protein | | − |
|  | *blr0652* |  | glutamine amidotransferase | | − |
|  | *blr0653* |  | phosphoribosylformino-5-aminoimidazole carboxamide ribotide isomerase | | − |
|  | *blr0654* | *hisF* | imidazole glycerol phosphate synthase subunit HisF | | -7.6 |
|  | *blr0655* | *hisE* | phosphoribosyl-ATP pyrophosphatase | | -2.6 |
|  | *blr0656* |  | pantothenate kinase | | − |
| *bll0781* |  |  | tRNA pseudouridine 55 synthase | | − |
|  | *bsl0780* | *rpsO* | 30S ribosomal protein S15 | | -10.0 |
| *blr0806* |  |  | hypothetical protein | | -7.6 |
| *bll0816* |  |  | hypothetical protein | | -8.6 |
| *bll0818* |  |  | hypothetical protein | | -20.7 |
| *bsr0858* |  |  | hypothetical protein | | -11.8 |
| *bsr0859* |  |  | hypothetical protein bsr0859 | | -8.0 |
| *bll0886* |  |  | ABC transporter ATP-binding protein | | -2.2 |
|  | *bll0885* |  | ABC transporter ATP-binding protein | | − |
|  | *bll0884* |  | ABC transporter permease protein | | − |
|  | *bll0883* |  | ABC transporter permease protein | | -10.4 |
| *bll0887* |  |  | ABC transporter substrate-binding protein | | -8.5 |
| *bll0892* |  |  | hypothetical protein | | -5.2 |
| *bll0905* |  | *regS* | two-component sensor histidine kinase | | − |
|  | *bll0904* | *regR* | two-component response regulator | | -91.7 |
| *blr0918* |  |  | ABC transporter permease protein | | − |
|  | *blr0919* |  | ABC transporter ATP-binding protein | | − |
|  | *blr0920* |  | hypothetical protein | | -5.4 |
| *blr0925* |  | *pcaF* | acetyl-CoA acetyltransferase | | -6.4 |
| *bsr0948* |  | *rpmF* | 50S ribosomal protein L32 | | -7.4 |
| *bsl0950* |  |  | hypothetical protein | | -5.3 |
| *blr1170* |  | *coxB* | cytochrome *c* oxidase subunit II | | − |
|  | *blr1171* | *coxA* | cytochrome *c* oxidase subunit I | | − |
|  | *blr1172* | *coxE* | putative heme *o* synthase | | − |
|  | *bsr1173* | *coxF* | CoxF protein | | − |
|  | *blr1174* | *coxG* | cytochrome *c* oxidase assembly protein | | -3.0 |
|  | *blr1175* | *coxC* | cytochrome *c* oxidase subunit III | | -5.5 |
| *bll1235* |  | *cysD* | O-acetylhomoserine sulfhydrylase | | -5.7 |
|  | *bll1234* |  | putative hydolase | | − |
| *blr1263* |  |  | unknown protein | | − |
|  | *blr1264* |  | hypothetical protein | | -6.4 |
|  | *blr1265* |  | hypothetical protein | | -6.3 |
| *bll1285* |  |  | hypothetical protein | | -151.1 |
| *blr1289* |  |  | hypothetical protein | | -8.1 |
| *bll1320* |  |  | probable penicillin-binding protein | | -5.3 |
| *blr1377* |  | *etfS* | electron transfer flavoprotein beta subunit | | -4.7 |
|  | *blr1378* | *etfL* | electron transfer flavoprotein large subunit | | -9.7 |
| *bll1385* |  | *pcaC* | putative gamma carboxymuconolactone decarboxylase protein | | -16.8 |
| *blr1424* |  |  | ABC transporter substrate-binding protein | | -2.6 |
|  | *blr1425* |  | ABC transporter substrate-binding protein | | -4.1 |
|  | *blr1426* |  | ABC transporter permease protein | | -5.1 |
|  | *blr1427* |  | ABC transporter permease protein | | -3.0 |
| *blr1429* |  |  | hypothetical protein | | -8.1 |
| *bll1464* |  |  | hypothetical protein | | -11.8 |
| *bll1467* |  |  | hypothetical protein | | -5.5 |
|  | *bll1466* |  | hypothetical protein | | -11.9 |
| *blr1468* |  |  | hypothetical protein | | -26.1 |
|  | *blr1469* |  | hypothetical protein | | -22.3 |
| *bsl1473* |  |  | hypothetical protein | | -14.9 |
| *bsl1507* |  | *rpmE* | 50S ribosomal protein L31 | | -13.7 |
| *blr1515* |  | *acrA* | RND multidrug efflux membrane permease | | -17.3 |
|  | *blr1516* | *acrB* | RND multidrug efflux transporter | | -13.4 |
| *bsl1589* |  |  | hypothetical protein | | -5.7 |
| *bsr1590* |  |  | hypothetical protein | | -6.7 |
| *blr1617* |  |  | conjugal transfer protein | | − |
|  | *blr1618* |  | probable conjugal transfer protein | | − |
|  | *blr1619* |  | conjugal transfer protein | | − |
|  | *blr1620* |  | conjugal transfer protein | | − |
|  | *bsr1621* |  | hypothetical protein | | -12.5 |
| *bsl1637* |  |  | unknown protein | | − |
|  | *bll1636* |  | hypothetical protein | | -6.0 |
| *bll1766* |  |  | outer membrane protein | | -10.2 |
| *bll1791* |  |  | hypothetical protein | | -5.5 |
| *blr1988* |  |  | unknown protein | | − |
|  | *blr1989* |  | unknown protein | | − |
|  | *blr1990* |  | hypothetical protein | | − |
|  | *blr1991* |  | hypothetical protein | | -5.4 |
| *blr2036^g^* |  | *fixR* | oxidoreductase | | -6.3 |
|  | *blr2037* | *nifA* | nif-specific regulatory protein | | -7.7 |
| *bsl2064* |  |  | hypothetical protein | | -5.3 |
| *bll2087* |  |  | hypothetical protein | | -5.2 |
| *bsl2212* |  |  | hypothetical protein | | -6.8 |
|  | *bll2211* | *copB* | copper tolerance protein | | -15.2 |
|  | *bll2210* | *copA* | multicopper oxidase | | -10.7 |
|  | *bll2209* | *copC* | copper tolerance protein | | -16.1 |
|  | *bll2208* |  | hypothetical protein | | -9.2 |
| *blr2351* |  |  | hypothetical protein | | -7.0 |
| *bll2388* |  | *cy_2_* | cytochrome *c_2_* | | -8.3 |
| *blr2405* |  | *fbp* | peptidylprolyl isomerase | | -5.2 |
| *bsl2407* |  |  | hypothetical protein | | -7.8 |
| *bll2462* |  |  | hypothetical protein | | -6.0 |
| *bll2465* |  |  | MoxR family protein | | -7.5 |
|  | *bll2464* |  | hypothetical protein | | -4.6 |
|  | *bll2463* |  | hypothetical protein | | -6.6 |
| *blr2501* |  |  | hypothetical protein | | -15.1 |
| *blr2505* |  |  | hypothetical protein | | -11.0 |
| *bsl2596* |  |  | hypothetical protein | | -7.8 |
| *bsl2602* |  |  | hypothetical protein | | -9.6 |
| *blr2694* |  |  | VirG-like two component response regulator | | -12.4 |
| *bll2737* |  |  | oxidoreductase with iron-sulfur subunit | | -14.2 |
|  | *bll2736* |  | putative aldehyde dehydrogenase protein | | -10.3 |
| *blr2787* |  |  | hypothetical protein | | -5.8 |
|  | *bsr2788* |  | unknown protein | | − |
| *blr2806^h^* |  |  | nitrite extrusion protein | | -10.3 |
|  | *blr2807* | *bjgb* | probable bacterial hemoglobin | | -4.0 |
|  | *blr2808* |  | putative FAD and NAD(P)H-binding reductase protein | | -9.2 |
|  | *blr2809* | *nasA* | nitrate reductase large subunit | | -5.5 |
| *blr2811* |  |  | hypothetical protein | | -5.0 |
| *bll2850* |  |  | probable 6-phosphofructokinase | | − |
|  | *bll2849* |  | hypothetical protein | | -8.4 |
| *bll2876* |  |  | ABC transporter permease protein | | -7.9 |
|  | *bll2875* |  | ABC transporter permease protein | | − |
|  | *bll2874* |  | ABC transporter ATP-binding protein | | − |
|  | *bll2873* |  | ABC transporter ATP-binding protein | | − |
| *blr2928* |  |  | oxidoreductase | | − |
|  | *blr2929* |  | hypothetical protein | | -10.2 |
|  | *blr2930* |  | hypothetical protein | | − |
| *bll3037* |  |  | hypothetical protein | | -11.9 |
| *bll3108* |  |  | hypothetical protein | | -6.2 |
| *blr3169* |  |  | hypothetical protein | | -38.3 |
| *blr3212* |  | *norE* | nitric oxide reductase subunit E | | -13.6 |
|  | *bsr3213* |  | hypothetical protein | | -10.8 |
| *blr3214* |  | *norC* | nitric oxide reductase subunit C | | -5.2 |
|  | *blr3215* | *norB* | nitric oxide reductase subunit B | | -4.9 |
|  | *blr3216* | *norQ* | NorQ protein | | -7.2 |
|  | *blr3217* | *norD* | NorD protein | | -11.3 |
| *blr3218* |  |  | putative hydrolase phosphatase protein | | -5.8 |
|  | *blr3219* |  | probable transcriptional regulator | | -4.5 |
| *blr3261* |  | *cobW* | cobalamin synthesis protein | | − |
|  | *blr3262* |  | hypothetical protein | | − |
|  | *blr3263* | *cobN* | cobaltochelatase | | -2.2 |
|  | *blr3264* |  | unknown protein | | − |
|  | *blr3265* | *cobH* | precorrin isomerase | | − |
|  | *blr3266* | *cobI* | precorrin-2 C20 methyltransferase | | -16.4 |
|  | *blr3267* | *cobJ* | precorrin-3B C17-methyltransferase | | − |
| *bll3466* |  | *fixK* | transcriptional regulator FixK | | -5.2 |
| *blr3555* |  |  | probable ferrichrome receptor precursor | | − |
|  | *bsr3556* |  | hypothetical protein | | -9.2 |
| *bll3592* |  |  | hypothetical protein | | -11.1 |
| *bll3717* |  | *lipA* | lipoyl synthase | | -5.8 |
| *bll3765* |  |  | glutamine amidotransferase | | -6.9 |
|  | *bll3764* |  | hypothetical protein | | -6.1 |
| *bll3768* |  |  | hypothetical protein | | -6.6 |
| *blr3769* |  |  | hypothetical protein | | -7.7 |
|  | *blr3770* |  | hypothetical protein | | -38.0 |
| *blr3771* |  |  | hypothetical protein | | -16.0 |
| *bll3817* |  |  | hypothetical protein | | -7.6 |
|  | *bll3816* |  | putative sulfur-regulated protein | | − |
| *blr3860* |  |  | hypothetical protein | | -21.5 |
| *blr3904* |  |  | probable iron transport protein | | -12.9 |
|  | *blr3905* |  | putative hydroxylase | | -12.4 |
|  | *blr3906* | *exbB* | biopolymer transport protein | | -13.6 |
|  | *blr3907* |  | biopolymer transport protein | | -26.6 |
|  | *blr3908* |  | hypothetical protein | | -8.1 |
| *bsl3938* |  |  | putative biotinylated protein | | -10.6 |
| *bsl4014* |  |  | hypothetical protein | | -5.6 |
| *blr4046* |  |  | hypothetical protein | | -7.8 |
| *bsr4099* |  |  | unknown protein | | − |
|  | *blr4100* |  | hypothetical protein | | -16.4 |
| *blr4119* |  | *ndk* | nucleoside diphosphate kinase | | -9.9 |
| *bll4130* |  |  | transcriptional regulatory protein | | -19.1 |
| *blr4156* |  |  | acetylornithine deacetylase | | -6.0 |
| *bll4168* |  |  | unknown protein | | − |
|  | *bsl4167* |  | putative glutamine synthetase translation inhibitor | | -6.8 |
| *blr4182* |  |  | hypothetical protein | | -28.2 |
| *bll4218* |  |  | hypothetical protein | | -26.0 |
| *bll4228* |  |  | putative ethidium resistance protein | | -8.1 |
|  | *bll4227* |  | transcriptional regulatory protein TetR family | | − |
| *bll4247* |  |  | hypothetical protein | | -5.2 |
| *bll4252* |  |  | putative hydrolase | | -5.1 |
| *blr4257* |  |  | putative hydrolase | | -3.9 |
|  | *bsr4258* |  | hypothetical protein | | − |
|  | *blr4259* |  | hypothetical protein | | -2.8 |
|  | *blr4260* |  | hypothetical protein | | -12.5 |
|  | *blr4261* |  | hypothetical protein | | -3.2 |
|  | *blr4262* |  | hypothetical protein | | -2.0 |
|  | *blr4263* |  | hypothetical protein | | − |
|  | *blr4264* |  | putative adenylate cyclase protein | | − |
| *bll4291* |  | *accB* | biotin carboxyl carrier protein subunit of acetyl-CoA carboxylasen | | -5.6 |
|  | *bll4290* |  | biotin carboxylase subunit of acetyl-CoA carboxylase | | − |
| *bll4292* |  | *aroQ* | 3-dehydroquinate dehydratase | | -19.6 |
| *bll4294* |  |  | outer membrane protein | | -5.5 |
| *blr4297* |  |  | hypothetical protein | | -6.0 |
| *bll4399* |  | *gloA* | lactoylglutathione lyase | | -5.1 |
| *bsr4408* |  |  | hypothetical protein | | -9.5 |
| *bll4412* |  |  | hypothetical protein | | -7.6 |
| *blr4416* |  |  | hypothetical protein | | -6.2 |
| *bsl4437* |  |  | hypothetical protein | | -10.1 |
| *blr4438* |  |  | hypothetical protein | | -5.9 |
|  | *blr4439* |  | penicillin binding protein | | -4.4 |
| *blr4442* |  |  | dolichol-phosphate mannosyltransferase | | − |
|  | *blr4443* |  | hypothetical protein | | -5.3 |
| *blr4505* |  |  | hypothetical protein | | -6.0 |
| *bll4579* |  |  | hypothetical protein | | -13.4 |
| *bll4583* |  | *qor* | quinone oxidoreductase | | -5.2 |
| *bll4589* |  |  | hypothetical protein | | -7.4 |
| *blr4723* |  |  | hypothetical protein | | -5.5 |
| *bll4736* |  |  | preprotein tranlocase protein | | -5.5 |
| *bll4819* |  |  | hypothetical protein | | -5.1 |
|  | *bll4818* |  | hypothetical protein | | − |
|  | *bll4817* |  | hypothetical protein | | -4.5 |
|  | *bll4816* |  | unknown protein | | − |
|  | *bll4815* |  | hypothetical protein | | -4.7 |
|  | *bll4814* |  | unknown protein | | − |
| *blr4839* |  | *gltA* | citrate synthase | | -8.0 |
| *bll4867* |  |  | putative outer-membrane immunogenic protein precursor | | -13.0 |
| *bll4896* |  |  | ABC transporter substrate-binding protein | | -5.7 |
| *bll4906* |  | *nuoL* | NADH dehydrogenase subunit L | | -4.4 |
|  | *bll4905* | *nuoM* | NADH dehydrogenase subunit M | | -4.0 |
|  | *bll4904* | *nuoN* | NADH dehydrogenase subunit N | | -6.2 |
|  | *bll4903* |  | birA bifunctional protein | | − |
|  | *bll4902* |  | hypothetical protein | | − |
| *bll4908* |  | *nuoJ* | NADH dehydrogenase subunit J | | -11.8 |
|  | *bll4907* |  | NADH dehydrogenase kappa subunit | | -9.8 |
| *bll4919* |  | *nuoA* | NADH dehydrogenase alpha subunit | | -2.2 |
|  | *bll4918* | *nuoB* | NADH ubiqionone oxidoreductase chain B | | − |
|  | *bll4917* | *nuoC* | NADH dehydrogenase subunit C | | -3.1 |
|  | *bll4916* | *nouD* | NADH dehydrogenase delta subunit | | -3.6 |
|  | *bll4915* |  | hypothetical protein | | -3.0 |
|  | *bll4914* |  | ATP synthase subunit E | | -8.4 |
|  | *bsl4913* |  | hypothetical protein | | -2.4 |
|  | *bll4912* | *nuoF* | NADH ubiquinone oxidoreductase chain F | | -14.7 |
|  | *bll4911* | *nuoG* | NADH dehydrogenase gamma subunit | | -8.1 |
|  | *bll4910* | *nuoH* | NADH dehydrogenase subunit H | | -10.5 |
|  | *bll4909* | *nuoI* | NADH dehydrogenase subunit I | | -31.3 |
| *bll4920* |  |  | ferrichrome iron receptor | | -9.2 |
| *blr4930* |  |  | hypothetical protein | | -3.4 |
|  | *blr4931* |  | hypothetical protein | | -13.4 |
|  | *blr4932* |  | putative cation efflux system protein | | -22.0 |
|  | *blr4933* |  | probable cation efflux system protein | | -26.5 |
| *blr4934* |  |  | hypothetical protein | | -8.4 |
| *blr4935* |  |  | putative divalent cation resistant determinant protein C | | -33.0 |
|  | *blr4936* |  | putative cation efflux system protein | | -23.3 |
|  | *blr4937* |  | probable cation efflux system protein | | -8.3 |
| *bll4983* |  |  | hypothetical protein | | -6.9 |
| *blr4984* |  |  | transcriptional regulatory protein | | -6.8 |
| *bll4985* |  |  | hypothetical protein | | -11.4 |
| *bsl5034* |  |  | hypothetical protein | | -5.1 |
| *bll5070* |  | *smpB* | SsrA-binding protein | | -7.6 |
|  | *bll5069* | *bcpB* | BcpB protein | | -4.7 |
| *bll5081* |  |  | putative multidrug resistance protein | | − |
|  | *bll5080* |  | AcrB/AcrD/AcrF family protein | | -2.7 |
|  | *bll5079* |  | hypothetical protein | | -5.6 |
| *bll5167* |  |  | glutathione S-transferase | | -8.9 |
|  | *bll5166* |  | hypothetical protein | | − |
| *blr5292* |  |  | hypothetical protein | | -7.6 |
| *bll5324* |  |  | hypothetical protein | | -5.3 |
| *blr5341* |  |  | hypothetical protein | | -5.4 |
| *bll5373* |  |  | probable short-chain dehydrogenase | | -7.1 |
|  | *bll5372* |  | hypothetical protein | | -7.5 |
| *bll5412* |  | *rplJ* | 50S ribosomal Protein L10 | | -7.8 |
|  | *bll5411* |  | 50S ribosomal protein L7/L12 | | − |
| *blr5422* |  |  | hypothetical protein | | − |
|  | *blr5423* |  | probable dTDP-glucose-4.6 dehydratase | | − |
|  | *blr5424* |  | hypothetical protein | | − |
|  | *blr5425* |  | hypothetical protein | | − |
|  | *blr5426* | *tktB* | transketolase | | 4.6 |
|  | *blr5427* |  | hypothetical transketolase family protein | | − |
|  | *blr5428* |  | hypothetical protein | | − |
|  | *blr5429* |  | hypothetical protein | | − |
|  | *blr5430* |  | hypothetical protein | | -8.7 |
|  | *blr5431* |  | hypothetical protein | | -3.1 |
|  | *blr5432* |  | hypothetical protein | | − |
| *bll5475* |  |  | putative formate dehydrogenase | | -6.4 |
| *bll5477* |  |  | similar to formate dehydrogenase | | -8.4 |
|  | *bll5476* |  | formate dehydrogenase iron-sulfur subunit | | -7.6 |
| *blr5502* |  |  | hypothetical protein | | -10.5 |
| *bll5555* |  |  | hypothetical protein | | -13.2 |
| *blr5556* |  |  | hypothetical protein | | -7.7 |
| *bsl5585* |  |  | hypothetical protein | | -7.6 |
| *blr5601* |  |  | hypothetical protein | | − |
|  | *blr5602* |  | hippurate hydrolase | | -5.7 |
|  | *blr5603* |  | glutamyl-tRNA amidotransferase subunit A | | − |
| *bsr5670* |  |  | hypothetical protein | | -8.1 |
| *blr5693* |  |  | probable substrate-binding protein | | -7.7 |
| *blr5730* |  |  | hypothetical protein | | -9.3 |
| *bll5734* |  |  | ABC transporter nitrate-binding protein | | − |
|  | *bll5733* |  | nitrate ABC transporter permease protein | | − |
|  | *bll5732* |  | ABC transporter ATP-binding protein | | − |
|  | *bll5731* |  | cyanate hydratase | | -6.3 |
| *bll5918* |  |  | putative dehydrogenase | | − |
|  | *bll5917* |  | DegT/DnrJ/EryC1/StrS family protein | | -5.7 |
| *blr5933* |  |  | hypothetical protein | | -8.4 |
| *bll5984* |  |  | unknown protein | | -4.1 |
|  | *bll5983* |  | unknown protein | | -3.5 |
|  | *bll5982* |  | hypothetical protein | | -11.3 |
| *blr5985* |  |  | unknown protein | | -3.7 |
|  | *blr5986* |  | hypothetical protein | | -6.5 |
| *blr5994* |  |  | hypothetical protein | | -7.8 |
|  | *blr5995* | *neuA* | hypothetical protein | | -4.3 |
|  | *blr5996* | *ptmB* | posttranslational modification protein | | -3.3 |
|  | *blr5997* |  | short chain dehydrogenase | | − |
|  | *blr5998* |  | putative membrane protein | | − |
|  | *blr5999* |  | unknown protein | | -4.7 |
|  | *blr6000* |  | hypothetical protein | | -8.5 |
| *blr6158* |  |  | ABC transporter substrate-binding protein | | -17.2 |
| *blr6167* |  |  | hypothetical protein | | -5.6 |
| *bll6198* |  |  | hypothetical protein | | -5.6 |
| *blr6210* |  |  | hypothetical protein | | -6.3 |
| *bll6262* |  | *osmC* | probable osmotically inducible protein | | -5.4 |
|  | *bll6261* |  | hypothetical protein | | -5.7 |
|  | *bll6260* |  | peptide methionine sulfoxide reductase | | − |
| *bll6455* |  |  | ABC transporter substrate-binding protein | | -2.4 |
|  | *bll6454* |  | ABC transporter permease protein | | -2.8 |
|  | *bll6453* |  | ABC transporter ATP-binding protein | | -3.5 |
|  | *bll6452* | *acd* | acyl-CoA dehydrogenase | | -5.8 |
|  | *bll6451* |  | probable alkanesulfonate monooxygenase | | -9.5 |
|  | *bll6450* |  | probable substrate-binding protein | | -10.2 |
| *bll6486* |  |  | hypothetical protein | | -5.6 |
|  | *bll6485* |  | hypothetical protein | | − |
| *bll6513* |  |  | hypothetical protein | | -8.7 |
| *blr6563* |  |  | hypothetical protein | | -8.3 |
|  | *blr6564* |  | putative dihydroflavonol-4-reductase | | -3.5 |
|  | *blr6565* |  | hypothetical protein blr6565 | | -7.4 |
| *blr6659* |  | *thiC* | thiamine biosynthesis protein ThiC | | -8.3 |
| *blr6660* |  |  | hypothetical protein | | -7.7 |
| *bll6668* |  |  | hypothetical protein | | -13.1 |
| *blr6718* |  |  | hypothetical protein | | -6.9 |
| *bsl6734* |  |  | hypothetical protein | | -6.9 |
| *bll6756* |  |  | hypothetical protein | | − |
|  | *bll6755* |  | hypothetical protein | | -3.4 |
|  | *bll6754* |  | hypothetical protein | | -5.7 |
| *blr6766* |  |  | hypothetical protein | | -3.0 |
|  | *blr6767* |  | trehalose synthase | | -4.0 |
|  | *blr6768* | *glgB* | glycogen branching enzyme | | -3.6 |
|  | *blr6769* | *glgX* | glycogen debranching enzyme | | -5.4 |
|  | *blr6770* |  | alpha-amylase | | − |
|  | *blr6771* |  | probable glycosyl hydrolase | | − |
| *bll6799* |  |  | hypothetical protein | | -20.0 |
| *bll6995* |  |  | tRNA (5-methylaminomethyl-2-thiouridylate)-methyltransferase | | − |
|  | *bll6994* |  | putative phosphatidylethanolamine N-methyltransferase | | − |
|  | *bll6993* |  | hypothetical protein | | -5.3 |
| *bll7010* |  | *ssuD* | sulfonate monooxygenase | | − |
|  | *bll7009* |  | aliphatic sulfonate ABC transporter permease protein | | -6.5 |
|  | *bll7008* |  | aliphatic sulfonate ABC transporter ATP-binding protein | | -3.5 |
|  | *bll7007* |  | putative oxidoreductase | | − |
| *bll7075* |  |  | hypothetical protein | | -18.2 |
|  | *bll7074* |  | hypothetical protein | | -15.2 |
|  | *bll7073* | *exbB* | biopolymer transport protein | | -9.5 |
|  | *bll7072* | *exbD* | biopolymer transport protein | | -15.9 |
|  | *bll7071* | *tonB* | TonB protein | | -5.8 |
| *bll7076* |  | *hmuR* | hemin receptor precursor | | -19.7 |
| *blr7077* |  | *hmuT* | hemin ABC transporter hemin-binding protein | | -7.3 |
|  | *blr7078* | *hmuU* | hemin ABC transporter permease protein | | -16.5 |
|  | *blr7079* | *hmuV* | hemin ABC transporter ATP-binding protein | | -4.0 |
| *blr7094* |  |  | hypothetical protein blr7094 | | -6.5 |
| *bsr7117* |  | *rpsU* | 30S ribosomal protein S21 | | -11.2 |
| *blr7131* |  |  | hypothetical protein blr7131 | | -7.1 |
| *blr7296* |  |  | hypothetical protein blr7296 | | -8.0 |
| *blr7297* |  |  | hypothetical protein blr7297 | | -12.2 |
| *bll7311* |  |  | probable ArcD2 arginine/ornithine antiporter | | -12.5 |
|  | *bll7310* |  | arginine deiminase | | -15.2 |
| *bll7313* |  |  | RND efflux membrane fusion protein | | -5.0 |
|  | *bll7312* |  | AcrB/AcrD/AcrF family protein | | -7.4 |
| *blr7314* |  |  | hypothetical protein | | -93.5 |
|  | *blr7315* |  | hypothetical protein | | -14.1 |
|  | *bsr7316* |  | hypothetical protein | | − |
|  | *bsr7317* |  | hypothetical protein | | − |
|  | *blr7318* |  | unknown protein | | -2.1 |
|  | *blr7319* |  | hypothetical protein | | − |
|  | *blr7320* |  | hypothetical protein | | -3.5 |
| *blr7321* |  |  | hypothetical protein | | -97.1 |
| *bll7322* |  |  | hypothetical protein | | -17.6 |
| *blr7323* |  |  | probable ArcD1 arginine/ornithine antiporter | | -8.3 |
| *blr7324* |  |  | hypothetical protein | | -4.8 |
|  | *blr7325* |  | hypothetical protein | | -9.4 |
|  | *blr7326* |  | hypothetical protein | | -7.9 |
| *blr7327* |  |  | hypothetical protein | | -17.2 |
| *bsr7328* |  |  | hypothetical protein | | -46.7 |
| *blr7329* |  |  | putative multidrag resistance protein | | -5.9 |
|  | *blr7330* |  | AcrB/AcrD/AcrF family protein | | -2.9 |
| *bll7411* |  |  | hypothetical protein | | -6.4 |
| *bll7414* |  |  | translation elongation factor EF-G | | -7.0 |
| *blr7436* |  |  | hypothetical protein | | -5.9 |
| *blr7465* |  |  | hypothetical protein | | -6.9 |
| *blr7471* |  | *pgsA* | phosphatidylglycerophosphate synthase | | -3.1 |
|  | *bsr7472* | *moaD* | molybdopterin converting factor small subunit | | -18.9 |
|  | *blr7473* | *moaE* | molybdopterin converting factor. large subunit | | − |
|  | *blr7474* |  | hypothetical adenine-specific methylase | | − |
| *blr7484* |  |  | hypothetical zinc protease | | -3.0 |
|  | *blr7485* |  | hypothetical zinc protease | | -7.6 |
| *blr7560* |  | *dhlB* | 2-haloalkanoic acid dehalogenase | | -19.1 |
|  | *blr7561* |  | hypothetical protein | | -3.3 |
| *bll7562* |  |  | hypothetical protein | | -9.1 |
| *bsr7564* |  |  | hypothetical protein | | -14.1 |
| *bll7626* |  |  | hypothetical protein | | -27.4 |
| *bll7628* |  |  | hypothetical protein | | -8.2 |
|  | *bll7627* |  | hypothetical protein | | -10.7 |
| *blr7629* |  |  | hypothetical protein | | -26.7 |
|  | *blr7630* |  | probable decarboxylase | | -6.8 |
| *bsr7633* |  |  | hypothetical protein | | -5.3 |
| *bll7635* |  |  | hypothetical protein | | -8.2 |
| *bll7638* |  |  | putative cytochrome *c_6_* precursor | | -6.8 |
|  | *bll7637* |  | unknown protein | | − |
|  | *bll7636* |  | hypothetical protein | | − |
| *blr7694* |  |  | hypothetical protein | | -6.8 |
| *bll7774* |  | *sodF* | superoxide dismutase | | -9.3 |
| *bll7790* |  |  | hypothetical protein | | -10.3 |
| *bll7795* |  | *phyR* | two-component response regulator | | -10.9 |
| *bsr7796* |  | *nepR* | anti-sigma factor | | -6.5 |
|  | *blr7797* | *ecfG* | RNA polymerase ECF sigma factor (σ^EcfG^) | | -3.7 |
| *blr7887* |  |  | hypothetical protein | | -6.1 |
| *bll7908* |  |  | hypothetical protein | | − |
|  | *bll7907* |  | hypothetical protein | | -3.9 |
|  | *bll7906* |  | putative ferredoxin | | -10.3 |
| *bll7938* |  |  | hypothetical protein | | -5.3 |
| *blr8111* |  |  | hypothetical protein | | -9.2 |
| *blr8132* |  |  | RhtB family transporter | | -5.2 |
| *trnE-CUC* |  |  | tRNA-Glu(CTC) | | -8.5 |
| *trnH-GUG* |  |  | tRNA-His(GTG) | | -7.5 |
|  |  |  |  | |  |
| **Class 2 (upregulated the Δ*regR* strain)** | |  |  | |  |
| *bsl0170* |  |  | hypothetical protein | | 5.4 |
|  | *bsl0169* |  | hypothetical protein | | 2.8 |
| *blr0624* |  |  | hypothetical protein | | 7.1 |
| *bll0777* |  |  | transcriptional regulatory protein | | 6.1 |
|  | *bll0776* |  | hypothetical protein | | 3.4 |
| *blr0903* |  |  | hypothetical protein | | 6.3 |
| *blr0960* |  |  | 5'-methylthioadenosine phosphorylase | | 3.5 |
|  | *blr0961* |  | translation initiation factor IF-2B subunit alpha | | 5.7 |
| *blr1072* |  |  | hypothetical protein | | 8.1 |
| *bll1113* |  |  | methylated-DNA--protein-cysteine methyltransferase | | 7.5 |
|  | *bll1112* |  | transcriptional regulatory protein | | − |
| *bll1150* |  |  | transcriptional regulatory protein | | 7.3 |
| *blr2071* |  |  | similar to inosamine-phosphate amidinotransferas | | 5.5 |
| *bsr2110* |  |  | hypothetical protein | | 5.1 |
|  | *bsr2111* |  | hypothetical protein | | 4.0 |
| *blr2113* |  |  | hypothetical protein | | 4.4 |
|  | *blr2114* |  | hypothetical protein | | 5.5 |
|  | *blr2115* |  | hypothetical protein | | 2.8 |
| *bll2512* |  |  | transcriptional regulatory protein | | 6.7 |
| *blr2605* |  |  | putative short chain dehydrogenase | | 5.4 |
| *bsl2907* |  |  | probable ferredoxin | | 6.7 |
| *blr2921* |  |  | hypothetical protein | | 5.6 |
|  | *blr2922* |  | ABC transporter amino acid-binding protein | | − |
| *bll3077* |  |  | transcriptional regulatory protein | | 7.7 |
| *bll3426* |  |  | ABC transporter substrate-binding protein | | 7.8 |
| *bll3668* |  |  | transcriptional regulatory protein | | 7.3 |
| *blr3741* |  |  | hypothetical protein | | 5.8 |
|  | *blr3742* |  | probable multidrug-resistance related protein | | 2.8 |
| *bll3785* |  | *coxM* | cytochrome *c* oxidase | | 5.7 |
|  | *bll3784* | *coxN* | cytochrome *c* oxidase | | 3.7 |
|  | *bll3783* | *coxO* | cytochrome *c* oxidase | | 3.0 |
|  | *bll3782* | *coxP* | cytochrome *c* oxidase | | − |
|  | *bll3781* |  | hypothetical protein | | − |
| *blr3787* |  |  | hypothetical protein | | 5.0 |
| *blr3963* |  |  | transcriptional regulatory protein | | 9.0 |
| *bll4010* |  |  | transcriptional regulatory protein PadR-like | | 12.5 |
| *blr4080* |  |  | transcriptional regulatory protein | | 3.6 |
|  | *blr4081* |  | hypothetical protein | | 6.2 |
| *bll4221* |  |  | transcriptional regulatory protein | | 6.6 |
| *blr4222* |  |  | phenol 2-monooxygenase | | 6.0 |
| *bll4347* |  |  | hypothetical protein | | 6.0 |
| *blr4499* |  |  | hypothetical protein | | 5.8 |
| *blr4673* |  |  | hypothetical protein | | 6.5 |
| *bll4873* |  |  | hypothetical protein | | 6.0 |
| *bsr4956* |  |  | hypothetical protein | | 5.6 |
| *bll5010* |  |  | putative resolvase | | 5.1 |
| *bll5164* |  |  | hypothetical protein | | 5.8 |
| *bll5199* |  |  | hypothetical protein | | 5.1 |
| *bll5353* |  |  | hypothetical protein | | 5.3 |
|  | *bll5352* |  | hypothetical protein | | 6.5 |
| *blr5497* |  |  | transcriptional regulatory protein | | 6.2 |
| *bll5501* |  |  | hypothetical protein | | 7.0 |
| *blr5658* |  |  | putative avidin | | 6.7 |
| *blr5735* |  |  | transcriptional regulatory protein | | 6.6 |
| *blr5860* |  |  | transcriptional regulatory protein | | 9.9 |
| *bll5900* |  |  | hypothetical protein | | 7.0 |
| *bll6110* |  |  | hypothetical protein | | 8.2 |
| *bll6243* |  | *hutI* | imidazolone-5-propionate hydrolase | | 11.2 |
|  | *bll6242* | *hutH* | histidine ammonia-lyase | | 5.1 |
|  | *bll6241* | *hutU* | urocanate hydratase | | 3.7 |
| *blr6244* |  |  | atrazine chlorohydrolase | | 6.7 |
|  | *blr6245* |  | transcriptional regulatory protein | | 3.2 |
| *blr6338* |  |  | hypothetical protein | | 5.1 |
|  | *blr6339* | *hyfB* | NADH dehydrogenase subunit N | | 4.4 |
|  | *blr6340* | *hycC* | probable hydrogenlyase component | | − |
|  | *blr6341* |  | hypothetical protein | | − |
|  | *blr6342* | *hyfF* | probable hydrogenlyase component | | − |
|  | *blr6343* |  | probable hydrogenlyase component | | − |
|  | *blr6344* | *hycG* | probable hydrogenase-3 subunit G | | − |
| *bll6512* |  | *thyA* | thymidylate synthase | | 3.4 |
|  | *bll6511* |  | acetyltransferase | | 5.6 |
|  | *bll6510* | *folA* | dihydrofolate reductase | | − |
| *bll6537* |  |  | putative cytochrome P_450_ | | 7.2 |
| *blr7050* |  |  | hypothetical protein | | 11.2 |
| *blr7098* |  |  | transcriptional regulatory protein | | 6.5 |
| *bll7214* |  |  | hypothetical protein | | 7.1 |
| *bsr7390* |  |  | hypothetical protein | | 5.5 |
| *bsr7727* |  |  | hypothetical protein | | 5.8 |
| *blr7895* |  |  | hypothetical protein | | 6.3 |
| *trnN-GUU-2* |  |  | tRNA-Asn(GTT) | | 6.4 |
| *trnF-GAA-2* |  |  | tRNA-Phe(GAA) | | 5.4 |

^a^ The list is a subset of genes differentially expressed in the Δ*regR* strain compared with the wild type, both grown in anoxically in BMS medium.

^b^ Gene numbers are according to the Rhizobase (http://genome.kazusa.or.jp/rhizobase/).

^c^ Operon predictions were performed as described by Hauser et al. (2007) [22] and Mesa et al. (2008) [14].

^d^ Genes names as indicated in the EMBL-EBI database with modifications.

^e^ Protein description according to Kaneko et al. (2002) [67] with modifications.

^f^ Fold change of expression in the Δ*regR* strain in comparison with the wild type, both grown anoxically. (−) indicates no change within the threshold fold change range between +5 and -5.

^g^ *bll2036* and *bl2037* constitute an operon unit described by Thöny et al. (1987) [68].

^h^ *blr2806, blr2807, blr2808* and *blr2809* have been shown to belong to an operon (J. Cabrera and M.J. Delgado, unpublished results), and *blr2807* has been recently identified as a haemoglobin and named as *bjgb* [44].
